# Making clinical guidelines work for people with multiple long term conditions: analysis and recommendations from review of single condition guidelines

**DOI:** 10.1136/bmjmed-2025-001495

**Published:** 2026-02-23

**Authors:** Sara Pretorius, Victoria Bartle, Sue Bellass, Ella-Rose Blackburn, Helen Bold, Rachel Cooper, Heather Cordell, Felicity Evison, Ray Holding, Nicola Howe, Emily Lam, Tom Marshall, Fiona Matthews, Paolo Missier, Ewan R Pearson, Chris Plummer, Gillian Richards, Sian M Robinson, Elizabeth Sapey, Thomas Scharf, Mervyn Singer, Jana Suklan, Phakapan Walker, James Wason, Avan A Sayer, Miles D Witham

**Affiliations:** 1 NIHR HealthTech Research Centre in Diagnostic and Technology Evaluation, Newcastle upon Tyne, UK; 2 Patient Advisory Group, Admission Research Collaborative, UK-wide, UK; 3 AGE Research Group, Translational and Clinical Research Institute, Faculty of Medical Sciences, Newcastle University, Newcastle upon Tyne, UK; 4 NIHR Newcastle Biomedical Research Centre, Newcastle upon Tyne Hospitals NHS Foundation Trust, Cumbria Northumberland and Tyne and Wear NHS Foundation Trust and Faculty of Medical Sciences, Newcastle University, Newcastle upon Tyne, UK; 5 Population Health Sciences Institute, Faculty of Medical Sciences, Newcastle University, Newcastle upon Tyne, UK; 6 PIONEER Hub, University of Birmingham, Birmingham, UK; 7 Data Science Team, Research, Development, and Innovation, University Hospitals Birmingham NHS Foundation Trust, Birmingham, UK; 8 Institute of Applied Health Research, University of Birmingham, Birmingham, UK; 9 Clinical and Applied Health Research, University of Hull, Hull, UK; 10 School of Computer Science, University of Birmingham, Birmingham, UK; 11 Division of Population Health and Genomics, Ninewells Hospital and School of Medicine, University of Dundee, Dundee, UK; 12 Digital Services, Newcastle Upon Tyne Hospitals NHS Foundation Trust, Newcastle upon Tyne, UK; 13 Institute of Inflammation and Ageing, University of Birmingham, Birmingham, UK; 14 Bloomsbury Institute for Intensive Care Medicine, University College London, London, UK; 15 Biostatistics Research Group, Population Health Sciences Institute, Faculty of Medical Sciences, Newcastle University, Newcastle upon Tyne, UK

**Keywords:** Medicine, Health services, Guideline adherence, Health policy, Quality of health care

## Abstract

**Objectives:**

To evaluate how UK guidelines for individual health conditions consider coexisting multiple long term conditions and to propose improvements to guideline development processes so that guidelines appropriately account for and consider coexisting multiple long term conditions.

**Design:**

Analysis and recommendations from review of single condition guidelines.

**Setting:**

Clinical guidelines developed by the National Institute for Health and Care Excellence (NICE), UK, 1 January 2013 to 31 December 2024.

**Population:**

56 clinical guidelines developed by NICE covering a broad range of long term conditions.

**Main outcome measures:**

The extent to which guideline recommendations consider multiple long term conditions and coexisting conditions, distinguishing between concordant conditions (those affecting the same organ system as the index condition) and discordant conditions (those affecting different systems).

**Results:**

All but one of the NICE guidelines (n=55, 98%) included some advice on managing the index condition in the presence of coexisting conditions, and 50 (89%) guidelines offered general guidance on tailoring care. Only 11 (20%) guidelines, however, explicitly referred to multiple long term conditions, and none included a dedicated section on multiple long term conditions or how care should be adapted in this context. 19 (34%) guidelines featured sections looking at specific coexisting conditions or coexisting conditions generally. Coverage of coexisting conditions varied widely across categories of conditions, with mental health guidelines dealing with the most coexisting conditions (median 10, interquartile range (IQR) 4.5-14.75) in contrast with guidelines on cancer and eye disease covering the fewest conditions (median 3, 1-4.5; median 3, 2.25-2.75, respectively). Of the 397 possible concordant pairings, 120 (30%) were referenced, whereas of the 3859 possible discordant pairings, 259 (7%) were referenced, indicating greater coverage of same system combinations. Data on the composition of guideline committees showed wide variation in size, disciplinary diversity, inclusion of generalist clinicians (eg, general practitioners, general physicians, or others with no single specialty focus), and public contributors, although lived experience of multiple long term conditions was rarely specified.

**Conclusions:**

Despite widespread acknowledgement of coexisting or multiple long term conditions, NICE guidelines are predominantly condition specific and offer limited tailored support for the care of multiple long term conditions. Recommendations rarely considered common condition clusters or the cumulative effect of multiple long term conditions. Structured improvements, such as clearer guidance on adapting care, broader cross condition referencing, and more transparent inclusion of lived experience could enhance the relevance and usability of guidelines for clinicians managing patients with multiple long term conditions.

**WHAT IS ALREADY KNOWN ON THIS TOPIC**

- Clinical guidelines have been widely criticised for focusing on one condition, with limited consideration of multiple long term conditions
- Previous reviews showed that guidelines rarely consider multiple long term conditions explicitly, often overlook discordant coexisting conditions, and seldom incorporate patient preferences or treatment burden
- Although recommendations for improvement have been proposed, adoption of these recommendations in current UK guidelines is unclear

**WHAT THIS STUDY ADDS**

- This analysis mapped the breadth and depth of related content on multiple long term conditions for diverse conditions and guideline structures
- Explicit guidance on multiple long term conditions was rare, with gaps in cross condition referencing, limited attention to discordant coexisting conditions, and minimal integration of lived experience
- The findings suggest that many of the recommendations made in earlier literature have yet to be implemented at scale

**HOW THIS STUDY MIGHT AFFECT RESEARCH, PRACTICE, OR POLICY**

- The study highlights the need for more systematic integration of consideration of multiple long term conditions into the development of guidelines
- Efforts should be made to incorporate multiple long term conditions into evidence review processes, committee design, and guideline structure, to achieve actionable, patient centred guidance
- Designing studies that reflect the complexity of real world multimorbidity is important, as well as the need for more usable, context sensitive tools to guide care for people with multiple long term conditions

## Introduction

 Multiple long term conditions, also referred to as multimorbidity, have been defined as the simultaneous presence of two or more chronic health conditions.^1^ Multiple long term conditions pose a major challenge for modern healthcare systems, patients, and clinical practice.^2^,^4^ Globally, estimates of the prevalence of multiple long term conditions range from 15% to 43% in the adult population,^5 6^ depending on the population studied, how data were collected and recorded, and how many conditions were included. Evidence suggests that the incidence of multiple long term conditions increases with age, and more than half of individuals in the UK aged ≥65 years have multiple long term conditions.^7^ Most individuals with a long term condition have more than one condition.^8^

The burden of multiple long term conditions and the accompanying investigations and treatment affect physical and psychological wellbeing, quality of life, and social functioning. People with multiple long term conditions have higher rates for attendance at emergency departments, unplanned hospital admissions, extended hospital stays, clinical complications, and polypharmacy, and an increased risk of premature mortality.^9^,^14^ These individuals also report suboptimal coordination of care and lower satisfaction with health services.^15^,^17^ The risk of poor outcomes from multiple long term conditions increases further in the presence of socioeconomic deprivation, mental health conditions, and acute conditions, such as influenza and SARS-CoV-2 infection.^2 18^

Despite the high prevalence and considerable burden of multiple long term conditions, clinicians have reported a lack of appropriate guidance on how to make care decisions with and for people with multiple long term conditions.^19^,^22^ Although clinical guidelines have helped in making treatment more evidence based and standardised, guidelines typically focus on one condition.^20^ This focus may be in part because recommendations frequently come from research directed at the management of just one condition, which often excludes individuals with multiple long term conditions.^23^ Another contributing factor may be that healthcare systems are organised around individual organ systems, ^1^which can obscure the complex interactions between different conditions and body systems and result in a less holistic approach to patient care.^24^

Combining multiple guidelines for the management of a set of different single conditions has been shown to produce conflicting advice and treatment,^25^ and a high burden of care for people with multiple long term conditions.^26^ For clinicians, managing patients with multiple long term conditions can be challenging, particularly when no clear primary diagnosis exists, making it difficult to prioritise problems, determine causes, or decide on the need for hospital admission. Specialist teams may be hesitant to assume responsibility, causing delays and fragmented care.^27^ Coexisting conditions can also restrict treatment choices; for example, a pacemaker might be appropriate for severe arrhythmia, but less appropriate for a patient with advanced malignancy and limited life expectancy. In these cases, guidelines on single diseases offer insufficient direction.

The question of how clinical guidelines account for coexisting conditions and multiple long term conditions is not new. Reviews over the past two decades have found that most guidelines focus on one disease, seldom looking at multiple long term conditions explicitly, and rarely integrate patient preferences. A 2005 review of nine US guidelines found no adaptation for older adults with multiple conditions, with risks of drug interactions, high costs, and reduced quality of life.^28^ Similarly, a Canadian guideline review found that although some guidelines looked at one additional condition, few considered the complexity of managing multiple coexisting conditions.^29^ These findings are important because managing one condition in the presence of one additional condition is fundamentally different from caring for a patient with multiple interacting conditions. Other studies have reported that guidelines focus mainly on concordant conditions (those affecting the same organ system as the index condition) rather than discordant conditions (those affecting different systems).^30^ UK based studies have supported these findings, with one study from 2017 reporting that guidelines from the National Institute for Health and Care Excellence (NICE) rarely provided treatment recommendations that considered coexisting conditions, with limited attention to drug-drug interactions or to applying the evidence to older adults.^20^ A 2013 review of five NICE clinical guidelines^25^ found that the guidelines inconsistently dealt with patient centred care, coexisting conditions, and treatment burden, with the depression guideline being a notable exception for its detailed drug-disease interaction table.

Several authors have made explicit recommendations for improving clinical guidelines for people with multiple long term conditions. These include cross referencing other relevant guidelines to highlight synergies, conflicts, and high risk interactions, providing patient vignettes for common coexisting condition combinations and offering specific advice for older patients (eg, on drug choice and dosing).^25^ Broader proposed frameworks recommend that guideline development panels should include multidisciplinary expertise, take a patient centred approach that incorporates the values and priorities of people with multiple long term conditions, and explicitly look at the impact of multiple long term conditions on all aspects of disease management.^22 23^

Previous reviews have typically focused on a small number of guidelines or specific conditions. In this study, our aim was to expand on earlier work by examining a broader set of current clinical guidelines. We explored the extent to which current guidelines incorporated advice relevant to coexisting or multiple long term conditions, and the degree to which past research recommendations for guideline development were implemented in practice. Our aim was not to assume that every guideline should look at all possible coexisting conditions, but rather to assess whether guidelines systematically acknowledged multiple long term conditions where relevant and provided sufficient guidance to support clinicians in managing patients with coexisting or multiple long term conditions. Given the high prevalence of multiple long term conditions in clinical practice, we believe that all single disease guidelines should at least indicate how coexisting or multiple long term conditions are taken into account.

In this review, we looked at these key questions: to what extent are coexisting or multiple long term conditions considered in NICE single condition guidelines; nature and scope of recommendations about coexisting or multiple long term conditions; extent to which guidelines cover concordant and discordant conditions; structure and composition of guideline development committees; and identification of guidelines that exemplify good practice in guideline development. Based on the findings of the study, we provide our own recommendations on how guideline developers could better incorporate advice on modifying recommendations in the presence of coexisting or multiple long term conditions.

## Methods

This study is part of the ADMISSION collaborative,^4 31^ a programme funded by UK Research and Innovation and UK based National Institute for Health and Care Research (NIHR), aiming to improve care for people with multiple long term conditions admitted to hospital. Based on five UK academic centres, ADMISSION brings together clinical, data, social, and computational sciences in partnership with public contributors. This review forms part of the ADMISSION Care Pathway Analysis work package, which focuses on understanding how healthcare professionals make decisions about referrals, admissions, and care for people with multiple long term conditions in secondary care.

### Selection of conditions and guidelines

We conducted a review of guidelines applicable to current UK clinical practice for a previously identified set of 60 long term conditions optimised for use when undertaking research on multiple long term conditions in a hospital setting.^32^ Although designed for research on hospital based multiple long term conditions, these conditions are also common in other care settings. Online supplemental table 1 has a full list of the conditions used.

We focused on NICE guidelines as the primary source for this review because these guidelines represent the national standard for evidence based clinical practice in England, Wales, and Northern Ireland, and are widely used across the NHS. We identified relevant guidelines for each condition by searching the NICE website (https://www.nice.org.uk/guidance) with the condition name as a search term and reviewing the most up-to-date guideline available. We sought guidelines that most closely aligned with the descriptors for the 60 conditions in our selected list. No specific NICE guidelines, however, were available for 20 of the conditions in the list: Addison's disease, arrhythmia, chronic liver disease, chronic pancreatic disease, hyperplasia of the prostate, paralysis, solid organ cancers, metastatic cancers, anxiety, connective tissue disease, long term musculoskeletal problems, inflammatory bowel disease, peptic ulcer, end stage kidney disease, anaemia, vision impairment that cannot be corrected, hearing impairment that cannot be corrected, Ménière's disease, HIV/AIDS, and congenital and chromosomal disorders. For these conditions, we sought related NICE guidelines that dealt with overlapping clinical concerns. Online supplemental table 1 provides an overview of the NICE guidelines reviewed in this study, showing which guidelines were included and how the guideline related to the 60 long term conditions that we assessed.

In some cases, closely related conditions were used as substitutes. For example, guidelines on atrial fibrillation, cirrhosis, pancreatitis, tinnitus, spinal injury, lower urinary tract symptoms, and rheumatoid arthritis were included to represent arrhythmia, chronic liver disease, chronic pancreatic disease, hearing impairment that cannot be corrected, long term musculoskeletal problems, hyperplasia of the prostate, and connective tissue disease, respectively. Although no NICE guideline specifically focused on diagnosing or treating osteoporosis, we included the guideline on assessing the risk of fragility fracture because it closely relates to the care and prevention of osteoporosis. To reflect the breadth of some umbrella terms, multiple guidelines were included for three descriptors: solid organ cancers, anxiety, and vision impairment that cannot be corrected. For solid organ cancers, we selected guidelines for five common types: breast, prostate, bladder, lung, and colorectal. These particular cancers are among the most prevalent in clinical practice and span a range of organ systems. Including these cancers allowed us to capture diversity in guideline content while maintaining a manageable scope for review. For anxiety, we included guidance on both social anxiety disorder and generalised anxiety disorder. For vision impairment, guidelines for glaucoma and age related macular degeneration were included. Although no dedicated guidelines existed for paralysis, metastatic cancer, or peptic ulcer, these conditions were partially covered in guidance on stroke, site specific cancers, and gastro-oesophageal reflux disease or dyspepsia, which corresponded to conditions in the original list.

### Data extraction

In this review, we defined multiple long term conditions as the presence of two or more chronic health conditions in an individual, regardless of whether one was considered primary. This definition differs from coexisting conditions or comorbidities, which usually refer to additional conditions occurring alongside a specific index condition. Although related, multiple long term conditions adopt a whole patient perspective rather than a one disease focus. Because few guidelines explicitly use the term multiple long term conditions, we used references to coexisting conditions or comorbidities as a pragmatic proxy for identifying whether and how guidelines looked at the complexity typical of people with multiple long term conditions.

For each guideline, two investigators (E-RB and SP) extracted content referring to coexisting or multiple long term conditions, including: references to unspecified coexisting conditions (eg, consider other health problems) or multiple long term conditions; references to specific coexisting conditions (eg, avoid β blockers in asthma); and recommendations for diagnosing, treating, or managing coexisting conditions in the presence of the index condition, and managing the index condition with consideration of other coexisting or multiple long term conditions. Each recommendation was categorised by content and reviewed by a third researcher (NH) for accuracy. For each guideline, we recorded whether multiple long term conditions or coexisting conditions were explicitly mentioned; whether the focus was on unspecified conditions, specific conditions, or multiple conditions; which specific coexisting conditions were identified, where applicable; the nature of recommendations made about coexisting or multiple long term conditions; and whether a dedicated section dealt with these concerns. To examine how the composition of the committee influenced the way guidelines considered multiple long term conditions, we recorded the roles of all committee members, recording the number of public or patient contributors, the number with a generalist background (including primary care, acute medicine, geriatric medicine, and paediatric medicine), and the number of specialists in the organ system relevant to the guideline. We also recorded the total number of distinct disciplines represented on each committee.

### Data analysis

To examine the extent to which single disease clinical guidelines considered coexisting or multiple long term conditions, we conducted a structured review of extracted guideline data. A guideline matrix was created to visualise which coexisting conditions were referenced in each guideline and the recommendations made. Recommendations were classified into predefined types; each was assigned a unique colour. Guidelines were grouped by body system (eg, cardiovascular, respiratory, neurological). Online supplemental table 1 has a list of the body system categories. A condensed version of the matrix was developed to present information (for each guideline) on: index condition; total number of coexisting conditions covered; how many of these conditions were concordant; referenced coexisting conditions; body systems considered; types of recommendations made; whether recommendations were made about multiple long term conditions or multimorbidity specifically, and the nature of these recommendations; and whether specific sections were included about coexisting or multiple long term conditions and the types of recommendations included. Acronyms for long term conditions were standardised with a predefined key.

The analysis was based on the predefined list of 60 chronic conditions. Each guideline was reviewed for explicit references to any of the conditions on this list. During data extraction, additional coexisting conditions were also recorded if explicitly mentioned in the guidelines but not included in the original list (eg, obsessive-compulsive disorder). In some instances, guidelines referred to a body system rather than to a specific condition within that system (eg, pre-existing cardiovascular disease); these data were also captured. This process resulted in an expanded set of 77 coexisting conditions used for analysis. To assess the extent to which guidelines looked at combinations of index and coexisting conditions, we first calculated the total number of possible combinations of condition. This number was derived by multiplying the number of guidelines reviewed (56) by the total number of distinct coexisting conditions mentioned across all guidelines (77) and then subtracting the number of guidelines (56) to exclude same condition pairings, resulting in a theoretical maximum of 4256 combinations of index-coexisting condition. We then identified which of these combinations were explicitly covered in the guideline content.

Combinations were further classified as concordant if the index and coexisting conditions were grouped under the same organ system and discordant if they were not. To assess how frequently guidelines referenced concordant versus discordant coexisting conditions, we calculated proportions for each organ system category. This method indicated the proportion of all possible concordant or discordant combinations that were actually observed. The proportion of concordant references was defined as the number of concordant coexisting condition references within guidelines for a given organ system, divided by the total number of possible concordant combinations for that category. The denominator was calculated by multiplying the number of guidelines in that organ system by the number of concordant coexisting conditions (ie, conditions within the same organ system) that could be mentioned. Similarly, the proportion of discordant references was calculated by dividing the number of discordant coexisting condition references by the total number of possible discordant combinations. This denominator was derived by multiplying the number of guidelines in the organ system by the number of coexisting conditions from other organ systems that could be mentioned.

We calculated the frequency of references to coexisting conditions for all body systems by extracting counts of mentions to conditions associated with other body systems in each guideline. These counts were then aggregated for all guidelines for each index body system to produce total reference frequencies. To account for variation in the number of guidelines for each body system, reference counts were normalised by dividing the total number of references to non-index body systems by the number of guidelines available for the index body system. This approach produced a relative frequency measure for each body system pairing, enabling comparison across clinical areas with different guideline volumes. To examine the composition of guideline committees, we calculated the proportion of public or patient contributors, members with a background in general medicine, and members who were specialists in the organ system relevant to the guideline relative to the total size of the committee.

### Recommendations

The list of recommendations in box 1 was developed through consultation with author members of the ADMISSION patient advisory group. During initial review of the guidelines, the authors identified gaps or limitations in guidelines that emerged as they were analysed and compared with other more comprehensive guidelines, leading to suggestions of what the authors and members of the patient advisory group believe constitutes best practice for guideline development. This finding was supported by conclusions from the existing literature.^2225 28^,^30 33^ Recommendations for identifying and synthesising guidelines also came from our experience of conducting this guideline review. Interim results of this review were shared periodically with the wider ADMISSION patient advisory group (n=20), culminating in a focused discussion on the most appropriate way to develop guidelines for the care of individuals with multiple long term conditions. This consultation informed the refinement and finalisation of our recommendations (box 1).

Box 1 **Key aspects for guideline developers to consider in ensuring that guidelines are appropriate for people with multiple long term conditions**

Committee composition

- Ensure that people with multiple long term conditions are represented on guideline committees.*
- Ensure that sufficient numbers of clinicians with generalist expertise are represented on guideline committees.

Literature searching and evidence synthesis

- Identify common and important conditions that coexist with the index condition at an early stage in evidence synthesis.
- Search specifically for evidence collected in patients with coexisting or multiple long term conditions when undertaking literature reviews that underpin single condition guidelines.
- Search specifically for evidence on burden of care and burden of conditions, as well as overall quality of life.

General guideline structure and content

- Identify and highlight common and important conditions that coexist with the index condition; both concordant and discordant conditions should be highlighted.
- Include a specific section on how guidance should be modified in the presence of coexisting or multiple long term conditions.
- Include a specific section on how patient education and self-care advice should be modified in the presence of coexisting or multiple long term conditions.
- Include a specific section on how approaches to service organisation, care coordination, and access to care should be adapted for people with coexisting or multiple long term conditions.
- Consider the potentially adverse effects of polypharmacy in the development of guidelines.

Guidelines should provide specific advice on two aspects

- How coexisting or multiple long term conditions influence the index condition, including:
  - Presentation
  - Diagnosis
  - Treatment
  - Monitoring
- How the index condition affects coexisting or multiple long term conditions, including:
  - Presentation
  - Identification
  - Diagnosis
  - Treatment
  - Monitoring

*The role of people with multiple long term conditions should include identifying common coexisting conditions and highlighting concerns about polypharmacy.

### Patient and public involvement

Patients and members of the public were involved throughout the development of this research. The NIHR HealthTech Research Centre Insight Panel provided feedback on the overall project, including the research design and study materials. Members of the ADMISSION patient advisory group contributed specifically to the development of the recommendations and reviewed the draft guidance for guideline developers (box 1). Their input helped ensure that the recommendations reflected the priorities and lived experiences of people with multiple long term conditions and their carers, and supported the clarity, relevance, and real world applicability of the final outputs.

This study did not involve direct research participants, and therefore no individual results exist to disseminate in that context, but online supplemental file 2 has a lay summary of the findings. We plan to share our findings with relevant patient and public communities through, for example, patient advisory groups, public engagement events, newsletters, or specific organisations. We will also ensure wider dissemination through academic conferences and stakeholder networks to maximise impact. We will share public friendly summaries on the ADMISSION and NIHR HealthTech Research Centre in Diagnostic and Technology Evaluation websites, social media, and newsletters.

## Results

### Description of guidelines

We identified 56 NICE guidelines in total. The oldest guideline was for social anxiety disorder, published in May 2013, and the most recent was the guideline for endometriosis, published in April 2024.

### Inclusion of coexisting or multiple long term conditions

The complete guideline matrix summarising which coexisting conditions were referenced in each guideline is available from the Newcastle University Figshare data repository (https://doi.org/10.25405/data.ncl.29911853.v1). Online supplemental table 3 has a condensed version of this matrix. Of the 56 guidelines reviewed, 55 (98%) gave some guidance on diagnosis, treatment, or other management of the index condition in the presence of at least one specific coexisting condition, whereas 50 (89%) gave advice on adapting care in the presence of coexisting conditions generally. Only 11 guidelines (20%), however, made specific reference to multiple long term conditions and how the presence of multiple long term conditions should modify care. Four of these directed the reader to the NICE guideline, Multimorbidity: clinical assessment and management, for recommendations on how to optimise care for patients with multimorbid conditions. None of the reviewed guidelines included a specific section on multiple long term conditions and how to tailor care. Nineteen guidelines (34%) included dedicated sections looking at clinical practice considerations for unspecified or specified coexisting conditions. Two of these focused on tailoring care for patients with coexisting conditions without specifying particular conditions. In 16 guidelines (29%), these sections covered specific coexisting conditions or condition categories, such as mental health.

Table 1 summarises the main ways in which other long term conditions were taken into account in condition specific guidelines. Advice on treatment or management in the presence of coexisting conditions (either specified or unspecified) was the most common category of recommendation (54/56 guidelines, 96%). These recommendations typically focused on adjusting treatment for individual coexisting conditions or for coexisting conditions in general. For example, the venous thromboembolic diseases guideline^36^ states *“*When offering anticoagulation treatment, take into account comorbidities,” and the thyroid disease guideline^37^ advises “Consider starting levothyroxine at a dosage of 25 to 50 micrograms per day with titration for adults aged 65 and over and adults with a history of cardiovascular disease.” The guidelines*,* however, rarely considered clusters of coexisting conditions that might be commonly associated with the index condition. Recommendations for testing and diagnosing coexisting conditions alongside the index condition, offering information, treatment, or referral for managing these coexisting conditions, and signposting to other specific guidelines (or the NICE guideline on multimorbidity^38^) were also frequently observed, appearing in more than half of the guidelines. Mentioned in fewer than half of the guidelines surveyed were: recommendations on testing for the index condition in the presence of a specific coexisting condition; applying coordinated, multidisciplinary care for people with multiple long term conditions; the index condition increasing the risk of specific coexisting conditions; recognising how coexisting or multiple long term conditions affect the needs, symptoms, or treatment of the index condition; and recommendations on ensuring equitable access to care in the presence of coexisting conditions. Online supplemental table 4 has examples of recommendations for each recommendation type. The Newcastle University Figshare data repository (https://doi.org/10.25405/data.ncl.29911853.v1) has a complete set of recommendations related to coexisting conditions from the reviewed guidelines, grouped by recommendation type.

**Table 1:** Number of guidelines where coexisting or multiple long term conditions recommendations were given (n=56)

Recommendation	Specific to a particular coexisting condition*	Not specific to a particular coexisting condition†	Neither§
Recommendation on treatment or management tailored to coexisting or multiple long term conditions	50/56 (89)	46/56 (82)	2/56 (4)
Recommendation on testing or diagnosing coexisting condition in the presence of index condition	31/56 (55)	16/56 (29)	23/56 (41)
Recommendation to provide information about, treat, or refer for management of a coexisting condition	34/56 (61)	25/56 (45)	16/56 (29)
Refer to relevant NICE guideline for managing coexisting or multiple long term conditions	36/56 (64)	14/56 (25)	19/56 (34)
Test for condition when specific coexisting condition is present	10/56 (18)	2/56 (4)	45/56 (80)
Use coordinated, multidisciplinary care for patients with multiple long term conditions	9/56 (16)	8/56 (14)	45/56 (80)
Ensure equity in care and access for coexisting conditions	8/56 (14)	6/56 (11)	47/56 (84)
Condition increases risk of other coexisting conditions or poorer outcomes	11/56 (20)	0/56 (0)	45/56 (80)
Recognise how coexisting or multiple long term conditions affect needs, symptoms, or drug treatment for condition	9/56 (16)	5/56 (9)	48/56 (86)

Values are number/total number (%).

Categories are not mutually exclusive. One guideline can provide recommendations that relate to a specific coexisting condition (eg, stroke and cancer) and also include more general recommendations (eg, stroke in the presence of various coexisting conditions). Therefore, one guideline can be counted in both categories, meaning that the sum of the rows may not equal the total number of guidelines.

* Recommendation looks at a particular coexisting condition. For example, from coronary heart disease guideline: “For people whose condition is stable after an MI (myocardial infarction), calcium channel blockers may be used to treat hypertension and/or angina.”

† Recommendation makes a general reference to coexisting condition or conditions. From example, from autism: “When discussing and deciding on interventions with autistic adults, consider: the presence, nature, severity, and duration of any coexisting disorders.”

§ Number of guidelines where the recommendation type does not appear.

NICE, National Institute for Health and Care Excellence.

### Coexisting conditions considered in guidelines

**Figure 1:**
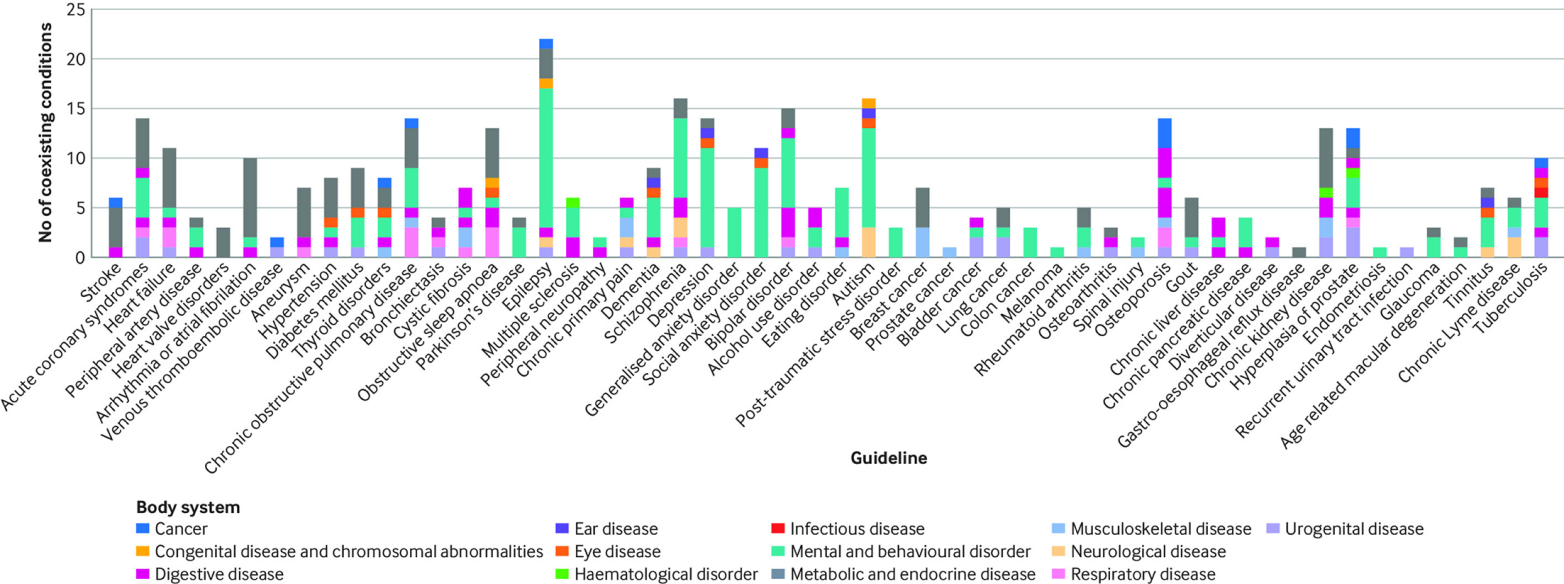
Number of coexisting conditions covered by each guideline, grouped by body system. Each bar represents one guideline, and colours correspond to the body system of coexisting conditions cited

Guidelines covered a median of six coexisting conditions, ranging from zero for guidelines on asthma and haematological cancers, to 22 for the epilepsy guideline. Figure 1 shows the total number of coexisting conditions covered by each guideline, reviewed by body system. We found notable variability across condition categories in the extent to which guidelines considered coexisting conditions. At one extreme, guidelines for cancer and eye disease each considered a median of three conditions (IQR 1-4.5 and 2.25-2.75), whereas mental health guidelines covered a median of 10 conditions (IQR 4.5-14.75). The most frequently mentioned coexisting conditions were diabetes mellitus and depression, each mentioned in 24 individual guidelines.

### Concordant versus discordant *c*onditions

**Figure 2:**
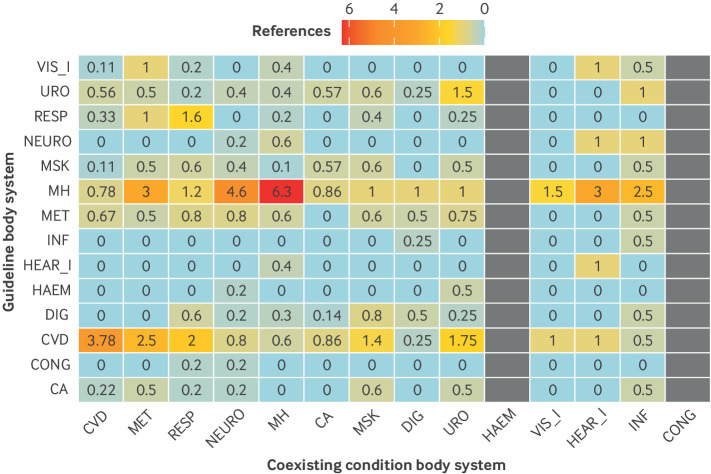
Normalised heatmap of guideline references to coexisting conditions for all body systems. Each cell represents the frequency of references to coexisting body systems (columns) within guidelines focused on a particular index body system (rows), adjusted for the number of guidelines for each body system. Darker shades indicate higher relative reference counts. CVD=cardiovascular, MET=metabolic-endocrine, RESP=respiratory, NEURO=neurological, MH=mental health, CA=cancer; MSK=musculoskeletal; DIG=digestive; URO=urogenital; HAEM=haematological; VIS_I=visual impairment; HEAR_I=hearing impairment; INF=chronic infection; CONG=congenital-chromosomal

Although 4256 pairings of index conditions with specific coexisting conditions were possible, only 379 (9%) of these were reflected in guideline recommendations. We found significant differences between condition categories in the proportion of condition combinations covered, ranging from 5/152 (3%) for eye disease to 101/760 (13%) for mental health. Of the 379 combinations of index condition-coexisting condition covered in guidelines, 120 (32%) were concordant combinations. Of the 397 possible concordant condition combinations, 120 (30%) were represented in guidelines, compared with 259 (7%) of 3859 possible discordant combinations (table 2). For most organ specific groups of conditions, concordant condition combinations were proportionately more likely to be mentioned than discordant condition combinations, with extremes represented by cancer (0% concordant *v* 4% discordant) and cardiovascular disease (44% concordant *v* 5% discordant). Table 2 has the full details. The normalised heatmap in figure 2 shows the variation in how guidelines referenced coexisting conditions for all body systems. Mental health guidelines showed broader cross system referencing, whereas cancer and eye disease guidelines had fewer references overall. Concordant references were more common in some categories, including cardiovascular disease and mental health, but discordant referencing patterns highlighted gaps in consideration of coexisting or multiple long term conditions across guideline types.

**Table 2:** Clustering of guidance according to concordant or discordant conditions

Body system	No of conditions in category	Median (IQR) No of coexisting conditions covered by guideline	Proportion of condition combinations covered (No/total No (%))			Concordant to discordant ratio
			Total conditions	Concordant conditions*	Discordant conditions†	
Cardiovascular	9	7 (4-10)	65/684 (10)	36/81 (44)	29/603 (5)	9
Metabolic and endocrine	2	8.5 (8.25-8.75)	17/152 (11)	2/6 (33)	15/146 (10)	3
Respiratory	5	7 (4-13)	38/380 (10)	8/25 (32)	30/355 (8)	4
Neurological	5	6 (4-6)	40/380 (11)	3/30 (10)	37/350 (11)	1
Mental health	10	10 (4.5 to −14.75)	101/760 (13)	58/150 (39)	43/610 (7)	5
Cancer	7	3 (1-4.5)	21/532 (4)	0/28 (0)	21/504 (4)	—
Musculoskeletal	5	5 (3-6)	30/380 (8)	3/30 (10)	27/350 (8)	1
Digestive	4	3 (1.75-4)	11/304 (4)	2/20 (10)	9/284 (3)	3
Urogenital	4	7 (1-13)	28/304 (9)	6/20 (30)	22/284 (8)	4
Eye disease	2	2.5 (2.25-2.75)	5/152 (3)	0/0 (0)	5/152 (3)	—
Ear disease	1	7	7/76 (9)	1/1 (100)	6/75 (8)	—
Chronic infection	2	8 (7-9)	16/152 (11)	1/6 (17)	15/146 (10)	2
Overall	56	6 (0-22)	379/4256 (9)	120/397 (30)	259/3859 (7)	4

Concordant combinations covered = total number of references to concordant conditions within body system guidelines ÷ (number of guidelines for body system × number of concordant coexisting conditions that could be referenced).

Discordant combinations covered = total number of references to discordant conditions within body system guidelines ÷ (number of guidelines for body system × number of discordant coexisting conditions that could be referenced).

* Conditions affecting the same organ system as the index condition covered by the guideline.

† Additional health conditions that do not belong to the same organ system as the index condition.

### Composition of guideline committee

For each NICE guideline, lists of committee members were available on the NICE website (https://www.nice.org.uk/guidance). The sizes of the guideline committees for the clinical guidelines varied greatly, ranging from a minimum of eight members for the chronic liver disease guideline to a maximum of 29 members for the dementia guideline. The median size of the guideline committee was 15 (IQR 12.75-18). Almost all of the guidelines (54, 96%) explicitly acknowledged the involvement of public or patient representatives in their development. For all committees, the proportion of committee members who were public or patient representatives ranged from 0% (obstructive sleep apnoea and chronic liver disease) to 23% (post-traumatic stress disorder and endometriosis), with a median of 15% (IQR 12-18%). Most guidelines referred to these members as lay members, patient or carer representatives, or service users, without clarifying whether individuals had the index condition or were service users in related areas. Only two guidelines explicitly identified public contributors as having lived experience: the dementia guideline (one patient with dementia) and the endometriosis guideline (two patients with endometriosis and three individuals with pregnancy complications).

The proportion of committee members with a generalist background ranged from 0% (haematological cancer) to 38% (chronic Lyme disease), with a median of 15% (IQR 11-20.5%). The proportion of members with a specialist interest in the index condition ranged from 6% (drug or alcohol misuse) to 67% (glaucoma), with a median of 40.5% (IQR 34.5-50%). The number of distinct disciplines represented for each committee ranged from two (glaucoma) to 14 (drug or alcohol abuse), with a median of 7 (IQR 5.75-9).

### Examples of good practice

Some of the guidelines that we reviewed were noteworthy for a more comprehensive approach to considering the effect of coexisting or multiple long term conditions on single condition management. Box 2 gives key features of four guidelines (epilepsy,^39^ chronic obstructive pulmonary disease,^40^ depression,^41^ and tuberculosis^42^). Each of the four guidelines included guidance on a greater range of coexisting conditions or gave more explicit attention to multiple long term conditions than most of the guidelines reviewed, and which together illustrate a range of approaches taken by different guideline developers.

Box 2 **National Institute for Health and Care Excellence (NICE) guidelines that explicitly consider coexisting or multiple long term conditions, and illustrative recommendations**

**Epilepsies in children, young people, and adults (NG217), published 27 April 2022** ^39^

Guideline strengths

- Considers numerous coexisting conditions, including discordant conditions.
- Dedicated sections on psychological, neurobehavioural, cognitive, and developmental conditions.
- Explicit consideration of multiple long term conditions.
- Emphasises patient education and empowerment.

Illustrative recommendations

- 4.1.2: Consider comorbidities when prescribing antiseizure drug treatments for older adults (eg, drug interactions, dosage adjustments, and multimorbidity informed approach).
- 4.5.1: Regular monitoring for adults with serious coexisting conditions (psychosocial, cognitive, and mental health).
- 9.1.2: Support coordinated care through multidisciplinary teams for epilepsy with mental health conditions or learning disabilities.

**Chronic obstructive pulmonary disease in over 16s: diagnosis and management (NG115), published 5 December 2018, last updated 26 July 2019** ^40^

Guideline strengths

- Considers a wide range of coexisting and discordant conditions.
- Dedicated sections on anxiety and depression.
- Explicit consideration of multiple long term conditions.

Illustrative recommendations

- 1.2.96: Multidisciplinary team delivery of chronic obstructive pulmonary disease care, covering coexisting conditions such as anxiety, depression, and physical health conditions.
- 1.2.82: Pulmonary rehabilitation exclusions for individuals with unstable angina, recent myocardial infarction, or inability to walk.
- 1.2.121: Patient information should explain how chronic obstructive pulmonary disease may affect other long term conditions (eg, hypertension, heart disease, and musculoskeletal issues).

**Depression in adults: treatment and management (NG222), published 29 June 2022** ^41^

Guideline strengths

- Considers numerous coexisting concordant and discordant conditions.
- Dedicated sections on specific coexisting conditions.
- Explicit consideration of multiple long term conditions.

Illustrative recommendations

- 1.4.29-30: Monitor lithium treatment with attention to physical health and drug treatment interactions.
- 1.11.1-4: Structured, multidisciplinary care for depression with coexisting personality disorders.
- 1.16.7-8: Collaborative care for older adults or those with chronic depression and major physical health problems.

**Tuberculosis (NG22), published 13 January 2016, last updated 16 February 2024** ^42^

Guideline strengths

- Considers a wide range of coexisting and discordant conditions.
- Dedicated sections on specific coexisting conditions.
- Advocates for multidisciplinary care.

Illustrative recommendations

- 1.3.7.14: Multidisciplinary team involvement for tuberculosis with coexisting conditions (eg, HIV, liver disease, diabetes, and substance misuse).
- 1.3.7.14: Specific guidance for managing tuberculosis alongside diabetes.
- 1.7.1.3 and 1.7.1.6: Enhanced case management for individuals with substance misuse to improve treatment adherence.

Illustrative recommendations are paraphrased to reflect the nature and intent of the original guidance, rather than quoted verbatim.

## Discussion

### Principal findings

This review of 56 NICE guidelines showed that although most guidelines made some provision for coexisting conditions, explicit consideration of multiple long term conditions was limited. Fifty five of 56 guidelines included at least some reference to coexisting conditions, but only 11 mentioned multiple long term conditions as a concept, and none had a dedicated section on how care should be adapted for people with multiple long term conditions. Coexisting conditions were most often referenced in relation to treatment decisions for individual conditions or broad generic statements, with much less frequent attention given to clusters of conditions, the cumulative effect of multiple long term conditions, or the need for integrated, patient centred care. These findings reflect earlier reports from 2011, which showed that guideline recommendations predominantly focused on patients with one concurrent condition and rarely looked at those with more than two conditions.^29^

Patterns of inclusion of coexisting conditions varied markedly across the condition categories. Mental health guidelines considered a broader range of coexisting conditions, including concordant and discordant conditions, whereas cancer guidelines had fewer references overall and were more likely to focus on concordant conditions. Patterns of inclusion may reflect differences in how multimorbidity is conceptualised across clinical domains. Mental health guidelines often originate from settings where coexisting conditions are the norm and care is more holistic, prompting broader consideration of concordant and discordant conditions. In contrast, cancer guidelines may be more specialist driven, where the focus tends to be narrower and condition specific, with multiple long term conditions considered mainly when treatment decisions are directly affected. These differences likely come from variations in the scope of the guideline, availability of the evidence, and clinical framing.

Our findings confirm earlier international reviews,^28^,^3035^ which consistently found that guidelines have been slow to deal with multiple long term conditions explicitly and tend to focus on concordant rather than discordant conditions. To some extent, concordant conditions would be expected to be referenced more frequently than discordant conditions, given their closer clinical relevance to the index condition. Our findings are consistent with this expectation. Discordant conditions, however, also warrant consideration, because treatments for one condition may complicate or conflict with the management of another, and the total number of conditions outside one organ system is usually greater than those affecting only that system. The relative neglect of discordant conditions in current guidelines may therefore contribute to challenges in providing coordinated care across specialty areas for people with multiple long term conditions. Although the relevance of multiple long term conditions varies by index condition, our findings suggest that many guidelines did not provide sufficient recognition of this problem, even when multiple long term conditions were highly prevalent (eg, chronic obstructive pulmonary disease and depression). This finding highlights a missed opportunity to support clinicians in delivering care that reflects the realities of clinical practice.

### Clinical guideline development since recommendations to improve relevance for multiple long term conditions

A framework was previously proposed for improving the relevance of clinical guidelines for people with multiple long term conditions, recommending broader clinical expertise, inclusion of patient and advocate perspectives, and explicit consideration of how coexisting conditions affect the effectiveness of treatment and outcomes.^22^ Inclusion of patient representatives in most guideline development groups suggests some attempt to incorporate patient and advocate perspectives, but whether these contributors reflect lived experience of the index condition or of multiple long term conditions more broadly is often unclear. This process limits the extent to which patient priorities and the realities of complex care are likely to inform recommendations. Although generalist clinicians, who often care for patients with multiple conditions, seemed to be less frequently represented than disease specific specialists, most committees included a mix of clinical expertise. This approach may reflect the condition specific remit of individual guidelines, although opportunities to incorporate broader perspectives on multiple long term conditions, treatment burden, and patient centred care could also be limited.

Part of our aim was to examine how single condition guidelines have responded to previous recommendations on dealing with coexisting or multiple long term conditions. Since many of these recommendations were made, NICE has published a dedicated multimorbidity guideline, which advises clinicians to consider multiple long term conditions when making decisions. This multimorbidity guideline recommends identifying patients who may benefit from a multimorbidity informed approach, reviewing treatment and disease burden, assessing patient goals and priorities, involving patients in decision making, and developing individualised management plans. Hence guideline developers are beginning to acknowledge the prevalence and clinical significance of multiple long term conditions, but our results suggest that these concerns have largely not been covered in single disease guidelines.

Nevertheless, we found some positive exemplars. Guidelines for epilepsy, chronic obstructive pulmonary disease, depression, and tuberculosis went beyond generic statements, incorporating detailed advice on coexisting conditions (including those outside of the indexed body system) and multiple long term conditions, and on providing multidisciplinary, patient centred care. These examples show that a stronger integration of considerations of multiple long term conditions into guideline development is both feasible and valuable.

### Key considerations for guideline development for multiple long term conditions

Taken together, our findings highlight a clear and ongoing gap between the complex realities of patient care and the predominantly single disease focus of current NICE guidelines. Building on previous research recommendations,^22 25 33^ and informed directly by our review, we propose four key areas for improving the way guidelines consider multiple long term conditions (box 1). Firstly, given that only two guidelines explicitly identified patient contributors as having lived experience of the index condition or multiple long term conditions, and that generalist clinicians were under-represented in many committees, committee composition should ensure the inclusion of people with multiple long term conditions and clinicians with generalist expertise. Secondly, because guideline recommendations often focused on adjusting treatment for individual coexisting conditions rather than considering clusters of conditions or the cumulative treatment burden, literature searching and evidence synthesis should explicitly target studies that include patients with coexisting or multiple long term conditions and assess outcomes such as treatment burden and quality of life. Thirdly, because none of the guidelines included a dedicated section on multiple long term conditions and few looked at discordant conditions, guideline structure should consistently include sections dealing with how care, patient education, and service organisation should be adapted in the presence of coexisting or multiple long term conditions, with attention to both concordant and discordant conditions. Finally, given that only a small proportion of the possible condition pairings were considered in recommendations, guidelines should provide specific, detailed advice on how multiple long term conditions modify the presentation, diagnosis, treatment, and monitoring of both the index condition and coexisting conditions. Incorporating these approaches into the development of guidelines would be a major step towards more realistic, patient centred recommendations for people with multiple long term conditions. In doing so, NICE could look at longstanding critiques of guideline development, reduce risks associated with polypharmacy and fragmented care, and support clinicians in delivering integrated, individualised care in line with the priorities and experiences of patients themselves.

Incorporating approaches informed by multiple long term conditions into the development of guidelines is essential for improving relevance and patient centredness, but raises important questions about clinical feasibility. Recent research highlights that current guidelines may be impossible to follow in full because of time constraints and workload pressures.^43^ Including detailed advice on coexisting or multiple long term conditions could risk further increasing the cognitive and administrative burden on clinicians unless carefully designed. To mitigate this risk, future guidelines should prioritise clarity, streamline decision pathways, and consider the time required to implement recommendations. For example, structured summaries, prioritisation tools, and decision aids could help clinicians navigate complex care scenarios more efficiently. By explicitly acknowledging workload implications and designing for usability, guideline developers can better support clinicians in delivering safe, integrated care without increasing the treatment burden.

Our recommendations highlight the need for guideline developers to consider coexisting conditions at the evidence synthesis stage. Our study, however, did not assess how these practices are currently undertaken. Future research could usefully examine how literature searches and synthesis methods can be adapted to capture evidence relevant to people with multiple long term conditions, including how the coexistence of conditions influences patient outcomes and experiences.

### Strengths and limitations of this study

Our analysis had several strengths. We used a predefined list of conditions, developed through a transparent process that was adapted from the results of a Delphi consensus process, that included patients and the public, and that reflected patient priorities rather than standard diagnostic labels. The range of conditions was broad relative to previous reviews, covering all major organ systems, and included both physical and mental health conditions. Also, our analysis examined a range of different types of guidance (eg, diagnosis, treatment, and healthcare system organisation) and specifically investigated whether guidelines had advice for people with multiple long term conditions rather than focusing on individual coexisting conditions. We also analysed the relation between the composition of the guideline committee and guideline advice, which likely will be of practical relevance for guideline committees and development processes.

We also acknowledge several limitations. Our choice of conditions did not always relate directly to the available guidelines, and many more guidelines and conditions existed that we did not include in our analysis. Nevertheless, we consider that we included sufficient guidelines to illustrate current gaps in the advice for people with multiple long term conditions. Guidelines differed in age, and multiple long term conditions as an entity has become a prominent consideration only in recent years. We chose a UK practice perspective, selecting NICE guidelines for our analysis. Our results, therefore, do not necessarily apply to guidelines developed in other jurisdictions, although previous work suggests that guidelines from the USA are also not configured to account for multiple long term conditions.^28^

Our decisions on classifying committee members as specialists or generalists had some degree of subjectivity, which limits the extent of our analyses on this topic. Also, publicly available data did not provide details on the specific roles or contributions of individual committee members, and did not indicate whether generalist clinicians had experience relevant to multimorbidity or had a central role in forming the recommendations. As such, the findings on the composition of the committees, and any inferences, should be interpreted with caution. We did not explicitly include references to polypharmacy as a distinct category of recommendations. Instead, this recommendation was incorporated under the broader category, recommendations on treatment or management in the presence of coexisting conditions. Given the prevalence of polypharmacy in individuals with multiple long term conditions and its associated risks, polypharmacy may have warranted its own category to ensure more focused attention. Nonetheless, we have highlighted the importance of polypharmacy and recommended that guideline developers explicitly consider polypharmacy when creating clinical guidelines.

### Guidelines as part of broader response to multiple long term conditions

Although this review highlights important opportunities to improve how guidelines consider multiple long term conditions, we recognise that the solution is not just adapting guidelines. Supporting high quality care for people with multiple long term conditions also requires changes in medical education, research, and health service structures, processes, and policy. For example, clinicians need training in managing complexity and uncertainty, health services need to provide integrated care pathways, and research must generate evidence that reflects populations with multiple long term conditions. We also acknowledge that generic recommendations to “consider multiple long term conditions” could be seen as unnecessary, because taking multiple long term conditions into account may be regarded as a core element of professional expertise. Our findings and previous research, however, suggest that without more explicit and practical guidance, clinicians may be challenged in reconciling conflicting recommendations, increasing the risk of fragmented or unsafe care. In this context, guidelines are important because they influence standards of care, inform service design, and provide a reference point for clinical decision making. Strengthening their relevance to people with multiple long term conditions should therefore be pursued in parallel with these broader system level changes.

### Conclusions

In this review, we showed that explicit consideration of multiple long term conditions is limited in NICE guidelines, despite longstanding requests for more integrated, patient centred approaches. References to coexisting conditions are common but often generic, with few guidelines offering detailed advice or reflecting the realities of complex care. Variation in condition areas and committee composition suggests missed opportunities to include lived experience and generalist expertise. Although positive examples show that more inclusive guidance is possible, stronger integration of considerations of multiple long term conditions is needed to support safer, more coordinated care.

## Supplementary material

10.1136/bmjmed-2025-001495 online supplemental table 1

10.1136/bmjmed-2025-001495 online supplemental file 1

10.1136/bmjmed-2025-001495 online supplemental table 2

10.1136/bmjmed-2025-001495 online supplemental table 3

## Data Availability

Data are available in a public, open access repository. Data are available upon reasonable request.
