# Supplementary material for: Making clinical guidelines work for people with multiple long term conditions: analysis and recommendations from review of single condition guidelines
Source: BMJ Med. 2026 Feb 23;5(1):e001495. doi: 10.1136/bmjmed-2025-001495 (PMC12933760; doi:10.1136/bmjmed-2025-001495)
Supplement: online supplemental table 1 [file bmjmed-5-1-s001.pdf]

Supplementary Table 1. List of 60 conditions used in guideline analysis (from Cooper et al [1]) and selected NICE guidelines for each condition

Body system (based on ICD-10 chapters)	Conditions for inclusion (n=60)	Selected NICE guideline	Year of Publication	Last Updated	NICE Reference
Cardiovascular disease	Stroke	Stroke and transient ischaemic attack in over 16s: diagnosis and initial management	01 May 2019	13 April 2022	NG128
	Coronary artery disease	Acute coronary syndromes	18 November 2020	*No Update*	NG185
	Heart failure	Chronic heart failure in adults: diagnosis and management	12 September 2018	03 September 2025	NG106
	Peripheral artery disease	Peripheral arterial disease: diagnosis and management	08 August 2012	11 December 2020	CG147
	Heart valve disorders	Heart valve disease presenting in adults: investigation and management	17 November 2021	*No Update*	NG208
	Arrhythmia	*No specific guideline: see atrial fibrillation*			
		Atrial fibrillation: diagnosis and management	27 April 2021	30 June 2021	NG196
	Venous thromboembolic disease	Venous thromboembolic diseases: diagnosis, management and thrombophilia testing	26 March 2020	02 August 2023	NG158
	Aneurysm	Abdominal aortic aneurysm: diagnosis and management	19 March 2020	*No Update*	NG156
	Hypertension	Hypertension in adults: diagnosis and management	28 August 2019	21 November 2023	NG136
Metabolic and endocrine disease	Diabetes mellitus	Type 2 diabetes in adults: management	02 December 2015	29 June 2022	NG28
	Addison's disease	*No Specific guideline*	-	-	-
	Thyroid disorders	Thyroid disease: assessment and management	20 November 2019	12 October 2023	NG145
Respiratory disease	Chronic obstructive pulmonary disease	Chronic obstructive pulmonary disease in over 16s: diagnosis and management	05 December 2018	26 July 2019	NG115
	Asthma	Asthma: diagnosis, monitoring and chronic asthma management	29 November 2017	22 March 2021	NG80
	Bronchiectasis	Bronchiectasis (non-cystic fibrosis), acute exacerbation: antimicrobial prescribing	18 December 2018	*No Update*	NG117
	Cystic Fibrosis	Cystic fibrosis: diagnosis and management	25 October 2017	*No Update*	NG78
	Obstructive sleep apnoea	Obstructive sleep apnoea/hypopnoea syndrome and obesity hypoventilation syndrome in over 16s	20 August 2021	*No Update*	NG202

Body system (based on ICD-10 chapters)	Conditions for inclusion (n=60)	Selected NICE guideline	Year of Publication	Last Updated	NICE Reference
Neurological disease	Parkinsons	Parkinson's disease in adults	19 July 2017	*No Update*	NG71
	Epilepsy	Epilepsies in children, young people and adults	27 April 2022	30 January 2025	NG217
	Multiple sclerosis	Multiple sclerosis in adults: management	22 June 2022	*No Update*	NG220
	Paralysis	*No specific guideline: see stroke, MS and spinal injury*	-	-	-
	Peripheral neuropathy	Neuropathic pain in adults: pharmacological management in non-specialist settings	20 November 2013	22 September 2020	CG173
	Chronic primary pain	Chronic pain (primary and secondary) in over 16s: assessment of all chronic pain and management of chronic primary pain	07 April 2021	*No Update*	NG193
Cancer	Solid organ cancers	*No specific guideline: see breast cancer, prostate cancer, bladder cancer, lung cancer and colorectal cancer*			
		Early and locally advanced breast cancer: diagnosis and management	18 July 2018	14 April 2025	NG101
		Prostate cancer: diagnosis and management	09 May 2019	15 December 2021	NG131
		Bladder cancer: diagnosis and management	25 February 2015	*No Update*	NG2
		Lung cancer: diagnosis and management	28 March 2019	08 March 2024	NG122
		Colorectal cancer	29 January 2020	15 December 2021	NG151
	Haematological cancers	Haematological cancers: improving outcomes	25 May 2016	*No Update*	NG47
	Metastatic cancers	*No Specific Guideline*	-	-	-
	Melanoma	Melanoma: assessment and management	29 July 2015	27 July 2022	NG14
Mental and behavioural disorder	Dementia	Dementia: assessment, management and support for people living with dementia and their carers	20 June 2018	*No Update*	NG97
	Schizophrenia	Psychosis and schizophrenia in adults: prevention and management	12 February 2014	18 March	CG178
	Depression	Depression in adults: treatment and management	29 June 2022	*No Update*	NG222

Body system (based on ICD-10 chapters)	Conditions for inclusion (n=60)	Selected NICE guideline	Year of Publication	Last Updated	NICE Reference
	Anxiety	*No specific guideline: see Generalised anxiety disorder and social anxiety disorder*			
		Generalised anxiety disorder and panic disorder in adults: management	26 January 2011	15 June 2020	CG113
		Social anxiety disorder: recognition, assessment and treatment	22 May 2013	*No Update*	CG159
	Bipolar disorder	Bipolar disorder: assessment and management	24 September 2014	*No Update*	CG185
	Drug or alcohol misuse	Alcohol-use disorders: diagnosis and management of physical complications	02 June 2010	12 April 2017	CG100
	Eating disorder	Eating disorders: recognition and treatment	23 May 2017	16 December 2020	NG69
	Autism	Autism spectrum disorder in adults: diagnosis and management	27 June 2012	14 June 2021	CG142
	Post-traumatic stress disorder	Post-traumatic stress disorder	05 December 2018	*No Update*	NG116
Musculoskeletal disease	Connective tissue disease	No specific *guideline: see Rheumatoid arthritis*			
		Rheumatoid arthritis in adults: management	11 July 2018	12 October 2020	NG100
	Osteoarthritis	Osteoarthritis in over 16s: diagnosis and management	19 October 2022	*No Update*	NG226
	Long-term musculoskeletal problems due to injury	*No specific guideline: see spinal injury*			
		Spinal injury: assessment and initial management	17 February 2016	*No Update*	NG41
	Osteoporosis	Osteoporosis: assessing the risk of fragility fracture	08 August 2012	07 February 2017	CG146
	Gout	Gout: diagnosis and management	09 June 2022	*No Update*	NG219
Digestive disease	Chronic liver disease	*No specific guideline: see cirrhosis*			
		Cirrhosis in over 16s: assessment and management	06 July 2016	08 September 2023	NG50
	Inflammatory bowel disease	*No specific guideline*	-	-	-
	Chronic pancreatic disease	*No specific guideline: see pancreatitis*			
		Pancreatitis	05 September 2018	16 December 2020	NG104
	Peptic ulcer	*No specific guideline. See gastro-oesophageal reflux disease and dyspepsia.*	-	-	-

Body system (based on ICD-10 chapters)	Conditions for inclusion (n=60)	Selected NICE guideline	Year of Publication	Last Updated	NICE Reference
	Diverticular disease	Diverticular disease: diagnosis and management	27 November 2019	*No Update*	NG147
	Gastro-oesophageal reflux disease	Gastro-oesophageal reflux disease and dyspepsia in adults: investigation and management	03 September 2014	18 October 2019	CG184
Urogenital disorder	Chronic kidney disease	Chronic kidney disease: assessment and management	25 August 2021	24 November 2021	NG203
	End-stage kidney disease	*No specific guideline.*	-	-	-
	Hyperplasia of the prostate	*No specific guideline: see lower urinary tract symptoms*			
		Lower urinary tract symptoms in men: management	23 May 2010	03 June 2015	CG97
	Endometriosis	Endometriosis: diagnosis and management	06 September 2017	11 November 2024	NG73
	Recurrent urinary tract infection	Urinary tract infection (recurrent): antimicrobial prescribing	31 October 2018	12 December 2024	NG112
Haematological disorder	Anaemia (including pernicious anaemia, sickle cell anaemia)	*No specific guideline.*	-	-	-
Eye disease	Vision impairment that cannot be corrected	*No specific guideline: see glaucoma and age-related macular degeneration*			
		Glaucoma: diagnosis and management	01 November 2017	26 January 2022	NG81
		Age-related macular degeneration	23 January 2018	*No update*	NG82
Ear disease	Hearing impairment that cannot be corrected	*No specific guideline: see tinnitus*			
		Tinnitus: assessment and management	11 March 2020	*No update.*	NG155
	Menieres disease	*No specific guideline.*	-	-	-
Infectious disease	HIV/AIDS	*No specific guideline.*	-	-	-
	Chronic Lyme Disease	Lyme disease	11 April 2018	17 October 2018	NG95
	Tuberculosis	Tuberculosis	13 January 2016	16 February 2024	NG33
Congenital disease	Congenital disease and chromosomal abnormalities	*No specific guideline.*	-	-	-

The 60 conditions were developed by the ADMISSION Research Collaborative; the list of conditions and the methods used to derive this condition list have been published previously [1]. These conditions were derived from a 2022 Delphi consensus exercise [2] and refined with input from experts and public contributors.

References:

1. Cooper, R., et al., *Rising to the challenge of defining and operationalising multimorbidity in a UK hospital setting: the ADMISSION research collaborative.* Eur Geriatr Med, 2024.
2. Ho, I.S.S., et al., *Measuring multimorbidity in research: Delphi consensus study.* BMJ Med, 2022. **1**(1): p. e000247.
