# Supplementary material for: Making clinical guidelines work for people with multiple long term conditions: analysis and recommendations from review of single condition guidelines
Source: BMJ Med. 2026 Feb 23;5(1):e001495. doi: 10.1136/bmjmed-2025-001495 (PMC12933760; doi:10.1136/bmjmed-2025-001495)
Supplement: online supplemental file 1 [file bmjmed-5-1-s002.pdf]

Supplementary File 2

Making clinical guidelines work for people living with multiple long-term conditions: Analysis and recommendations from a review of single-condition guidelines

Plain English Summary

Many people live with more than one long-term health condition at the same time. This is known as having multiple long-term conditions (MLTC) or multimorbidity. It is common, especially in older adults, and can make healthcare more complicated. People with MLTC often need to manage several treatments, attend multiple appointments, and follow advice from different specialists. This can affect their physical and mental health, quality of life, and satisfaction with care. It also increases the need for health services, leading to more hospital admissions, emergency visits, and medicines being prescribed.

Despite this, clinical guidelines, which support healthcare professionals in making decisions, usually focus on one condition at a time. This can lead to conflicting advice, uncertainty, and fragmented care for people living with MLTC. Clinicians have said they need better guidance on how to support people with MLTC, especially when conditions interact or when one treatment may worsen another condition.

In this review, we looked at 56 clinical guidelines published by the UK National Institute for Health and Care Excellence (NICE) written between 2013 and 2024 to identify any references to co-existing conditions or MLTC. We examined whether guidelines discussed conditions that affect the same part of the body or those that affect different body systems. We also reviewed the membership of the committees that developed each guideline, including how many members were generalists (such as GPs or clinicians who care for older people), how many were specialists, and whether people with lived experience of MLTC were involved in generating each guideline.

Most guidelines included some advice about caring for people who have other health problems and encouraged tailoring care to individual needs. However, only a few referred directly to MLTC, and none had a dedicated section on how to adapt care when several conditions are present. Mental health guidelines covered co-existing conditions more thoroughly than others, while cancer and eye disease guidelines covered coexisting conditions least thoroughly.

Guidelines were more likely to mention other health problems that affect the same part of the body, rather than those that affect other body parts. Most committees included patients or members of the public, but only two clearly said that these people had personal experience of the condition. Generalist clinicians were included in some committees, but there were fewer of them compared to specialists focused on a single area of medicine.

To help improve future guidelines, we worked with members of the ADMISSION Patient Advisory Group to develop several recommendations. These include involving people with lived experience of MLTC and generalist clinicians in guideline development; actively searching for research evidence about co-existing conditions; identifying common co-existing conditions clearly; including sections on how care should be adapted for people with MLTC; and addressing the risks of multiple medicines (polypharmacy) and interactions between conditions and treatments.

Our findings show that while awareness of MLTC is increasing, most current guidelines still provide limited practical advice for people with multiple conditions. We recommend clearer guidance on tailoring care and more meaningful involvement of people with lived experience in developing future guidelines.
