# Supplementary material for: Making clinical guidelines work for people with multiple long term conditions: analysis and recommendations from review of single condition guidelines
Source: BMJ Med. 2026 Feb 23;5(1):e001495. doi: 10.1136/bmjmed-2025-001495 (PMC12933760; doi:10.1136/bmjmed-2025-001495)
Supplement: online supplemental table 2 [file bmjmed-5-1-s003.pdf]

Supplementary Table 3 - Data on the recommendations around co-existing conditions and MLTC in single disease guidelines

Acronym Key for Long-Term Health Conditions

ADHD	Attention Deficit Hyperactivity Disorder	DM	Diabetes Mellitus	OP	Osteoporosis
AF	Arrhythmia/Atrial Fibrillation	END	Endometriosis	OSA	Obstructive Sleep Apnoea
AI	Automimmune Disease	EPI	Epilepsy	PAD	Peripheral Arterial Disease
ANA	Anaemia	FM	Fibromyalgia	PD	Parkinson's Disease
ANEU	Aneurysm	GORD	Gastro-oesophageal Reflux Disease	PN	Peripheral Neuropathy
ANX	Anxiety Disorder	GOUT	Gout	PSY	Psychosis (Not Specified)
ASD	Autism Spectrum Disorder	HAEM	Haematological disorder	PTSD	Post-Traumatic Stress Disorder
ASTH	Asthma	HEAR-I	Hearing impairment (Not Specified)	RA	Rheumatoid Arthritis
BPD	Bipolar Disorder	HEP-C	Hepatitis C	REN	Renal Dysfunction/Impairment
BRONC	Bronchiectasis	HF	Heart Failure	RESP	Respiratory/Lung Disease
CA	Cancer/Malignancy	HIV	HIV/AIDS	RUTI	Recurrent Urinary Tract Infections
CA-B	Cancer (breast)	HTN	Hypertention	SCZ	Schizophrenia
CA-H	Cancer (haematological)	HTN-O	Orthostatic Hypertension	SLE	Systemic Lupus Erythematosus
CA-L	Cancer (lung)	HVD	Heart Valve Disorder	STR	Stroke
CA-P	Cancer of the prostate	IBD	Irritable Bowel Disease	SUB	Drug or Alcohol Misuse
CF	Cystic Fibrosis	INF	Infection	SUI	Suicide risk
CHD	Coronary Heart Disease	LD	Learning Disability	THYR	Thyroid Disorders
CKD	Chronic Kidney Disease	HIV	HIV/AIDS	URO	Urogential disorder
CLD	Chronic Liver Disease	HTN	Hypertention	VIS-I	Vision Impairment
COG	Cognitive impairment	HVD	Heart Valve Disorder	VTD	Venous Thromboembolic Disease
CONG	Congenital/Chromosomal Abnormality	IBD	Irritable Bowel Disease		
COPD	Chronic Obstructive Pulmonary Disease	INF	Infection		
CPD	Chronic Pancreatic Disease	LD	Learning Disability		
CPP	Chronic Primary Pain	MSK	Musculoskeletal problem		
CVD	Cardiovascular Disease	NDD	Neurodevelopmental Disorder		
DEM	Dementia	NEURO	Neurological Disorder/Impairment		
DEP	Depression	OA	Osteoarthritis		
DIG	Digestive Disease	OCD	Obsessive Compulsive Disorder		

Categories of Clinical Guidance Related to Co-Existing Conditions and MLTC

Recommendations on treatment or management tailored to co-existing conditions/MLTC

Recommendation on testing/diagnosing co-existing condition/MLTC in the presence of index condition

Test for condition when specific co-existing condition is present

Recognise how co-existing conditions/MLTC affect needs, symptoms, or medication for condition

Condition increases risk of other co-existing conditions or poorer outcomes

Ensure equity in care and access for co-existing conditions

Recommendation to provide information about, treat or refer for management of co-existing condition/MLTC

Refer to relevant NICE guideline for managing co-existing conditions/MLTC

Use coordinated, multidisciplinary care for patients with MLTC

Organ/Body System	Guideline - Condition	No of co-existing conditions addressed	Specific co-existing conditions addressed	Body systems addressed	Number of concordant co-existing conditions	Recommodations around co-existing conditions		Recommendations around MLTC	Specific section for co-existing conditions/or MLTC
						Co-existing conditions (not specified)	Co-existing conditions (specified)		
						Recommendation type			
Cardiovascular disease	Stroke	6	AF, VTD, ANEU, HTN, DM, CA	CVD, MET, CA	4				
	Acute Coronary Syndromes	14	HF, AF, HTN, VTD, CVD, DM, COPD, DEP, ANX, LD, MH, GORD, CKD, REN	CVD, MET, RESP, MH, DIG, URO	5				
	Heart failure	11	CHD, HVD, PAD, AF, HTN, CVD, DM, COPD, RESP, DEP, CKD	CVD, MET, MSK, RESP, MH, URO	6				
	Peripheral artery disease	4	HTN, DM, DEP, ANX	CVD, MET, MH	1				
	Heart valve disorders	3	CHD, HF, AF	CVD	3				
	Arrhythmia / Atrial Fibrilation	10	STR, CHD, HF, HVD, PAD, VTD, HTN, CVD, DM, SUB	CVD, MET, MH	8				
	Venous thromboembolic disease	2	CA, REN	CA, URO	0				
	Aneurysm	7	CHD, PAD, HTN, CVD, DM, COPD, VTD	CVD, MET, RESP	5				
	Hypertension	8	CHD, HF, AF, CVD, DM, CKD, VIS-I, SUB	CVD, MET, URO, VIS-I, MH	4				
Metabolic and endocrine disease	Diabetes mellitus	9	HF, HTN, CVD, PN, DEP, LD, COG, CKD, VIS-I,	CVD, MH, URO, VIS-I,	0				
	Thyroid disorders	8	AF, CVD, DM, AI, DEP, ANX, VIS-I, CA	CVD, MET, MSK, MH, VIS-I, CA	2				
Respiratory disease	Chronic obstructive pulmonary disease	14	CHD, HF, HTN, CVD, DM, ASTH, BRONC, DEP, ANX, MH, COG, MSK, CA-L, RESP	CVD, MET, RESP, MH, MSK, CA	3				
	Asthma	0			0				
	Bronchiectasis	4	CVD, RESP, CLD, CKD	CVD, RESP, DIG, URO	1				
	Cystic fibrosis	7	DM, BRONC, MH, OA, CLD, CPD. MSK	MET, RESP, MH, MSK, DIG	1				
	Obstructive sleep apnoea	13	STR, HF, AF, HTN, CVD, DM, THYR, COPD, ASTH, RESP, SUB, VIS-I, CONG	CVD, MET, RESP, MH, VIS-I, CONG	3				

Organ/Body System	Guideline - Condition	No of co-existing conditions addressed	Specific co-existing conditions addressed	Body systems addressed	Number of concordant co-existing conditions	Recommodations around co-existing conditions		Recommendations around MLTC	Specific section for co-existing conditions/or MLTC
						Co-existing conditions (not specified)	Co-existing conditions (specified)		
						Recommendation type			
**Neurological disease**	Parkinson's Disease	4	DEM, DEP, MH, HTN-O	MH, CVD	1				
	Epilepsy	22	STR, AF, HTN, DM, PD, CA, SCZ, DEP, ANX, SUB, ASD, PTSD, LD, PSY, SUI, ADHD, OCD, MH, NDD, COG, REN, CONG	CVD, MET, NEURO, CA, MH, URO, CONG	1				
	Multiple sclerosis	6	DM, THYR, DEP, ANX, MH, ANA	MET, MH, HAEM	0				
	Peripheral neuropathy	2	MH, DM	MH, MET	0				
	Chronic primary pain	6	PN, SUB, RA, OA, IBD, END	NEURO, MH, MSK, DIG, URO	1				
**Mental health and behavioural disorder**	Dementia	9	HTN, DM, DEP, ANX, LD, VIS-I, HEAR-I, COG, PD	CVD, MET, MH, VIS-I, HEAR-I, NEURO	2				
	Schizophrenia	16	HTN, CVD, DM, RESP, PD, DEM, NEURO, DEP, ANX, SUB, PTSD, NDD, REN, MET, BPD, PSY	CVD, MET, RESP, NEURO, MH, NEURO, URO	7				
	Depression	14	ANX, DEM, ASD, LD, MH, NDD, COG, VIS-I, HEAR-I, BPD, PTSD, REN, CVD, PSY	MH, VIS-I, HEAR-I, URO, CVD	9				
	Generalised anxiety disorder	5	DEP, ANX, SUB, PTSD, MH	MH	5				
	Social anxiety disorder	11	DEP, ANX, SUB, ASD, LD, PSY, ADHD, MH, NDD, VIS-I, HEAR-I	MH, VIS-I, HEAR-I	9				
	Bipolar disorder (BPD)	15	HTN, CVD, DM, MET, RESP, ANX, SUB, LD, ADHD, MH, THYR, CLD, CKD, NDD, COG	CVD, MET, RESP, MH, DIG, URO	7				
	Alcohol Use Disorder	5	LD, COG, CLD, REN, CPD	MH, DIG, URO	2				
	Eating disorder	7	DM, DEP, ANX, SUB, OCD, MH, OA	MET, MH, MSK	5				
	Autism	16	EPI, CPP, NEURO, SCZ, DEP, ANX, SUB, LD, PSY, OCD, MH, NDD, VIS-I, HEAR-I, ADHD, CONG	NEURO, MH, VIS-I, HEAR-I, CONG	9				
	Post-traumatic stress disorder	3	DEP, SUB, MH	MH	3				

Organ/Body System	Guideline - Condition	No of co-existing conditions addressed	Specific co-existing conditions addressed	Body systems addressed	Number of concordant co-existing conditions	Recommodations around co-existing conditions: Co-existing conditions (not specified)	Recommodations around co-existing conditions: Co-existing conditions (specified)	Recommendations around MLTC	Specific section for co-existing conditions/or MLTC
						Recommendation type			
Cancer	Breast Cancer	7	CHD, HF, AF, HTN, RA, OA, FM	CVD, MSK	0				
	Prostate cancer	1	OA	MSK	0				
	Bladder cancer	4	COG, IBD, CKD, REN	MH, DIG, URO	0				
	Lung cancer	5	CHD, CVD, DEP, CKD, REN	CVD, MH, URO	0				
	Colon cancer	3	ANX, DEP, COG	MH	0				
	Haematological cancers	0			0				
	Melanoma	1	MH	MH	0				
Musculoskeletal disease	Rheumatoid arthritis	5	HTN, CVD, DEP, MH, OA	CVD, MH, MSK	1				
	Osteoarthritis	3	CVD, CLD, CKD	CVD, DIG, URO	0				
	Spinal injury	2	MH, OA	MH, MSK	1				
	Osteoporosis	14	CA-B, CA-P, THYR, MET, DM, IBD, CLD, CPD, RA, CA-H, CF, COPD, CKD, SUB	CA, MET, DIG, MSK, CA, RESP, URO, MH	1				
	Gout	6	STR, CHD, HTN, CVD, SUB, CKD	CVD, MH, URO	0				
Digestive disease	Chronic liver disease	4	DM, SUB, CLD, HEP-C	MET, MH, DIG, INF	1				
	Chronic pancreatic disease	4	DM, SUB, DEP, MH	MET, MH	0				
	Diverticular disease	2	CLD, CKD	DIG, URO	1				
	Gastro-oesophageal reflux disease	1	HTN	CVD	0				

Organ/Body System	Guideline - Condition	No of co-existing conditions addressed	Specific co-existing conditions addressed	Body systems addressed	Number of concordant co-existing conditions	Recommodations around co-existing conditions		Recommendations around MLTC	Specific section for co-existing conditions/or MLTC
						Co-existing conditions (not specified)	Co-existing conditions (specified)		
						Recommendation type			
Urogential disorder	Chronic kidney disease	13	HF, PAD, HTN, CVD, DM, SLE, GOUT, CKD, REN, ANA, AF, VTD, THYR	CVD, MET, MSK, URO, HAEM	2				
	Hyperplasia of the prostate	13	HF, DM, OSA, CA, DEP, LD, MH, CLD, CKD, REN, RUTI, ANA, CA-P	CVD, MET, RESP, CA, MH, DIG, URO, HAEM, CA	3				
	Endometriosis	1	COG	MH	0				
	Recurrent urinary tract infection	1	REN	URO	1				
Eye disease	Glaucoma	3	HTN,LD, COG	CVD, MH	0				
	Age-related macular degeneration	2	HTN, DEP	CVD, MH	0				
Ear disease	Tinnitus	7	STR, DEP, COG, HEAR-I, VIS-I,NEURO, MH	CVD, MH, HEAR-I, VIS-I, NEURO	1				
Chronic Infection	Chronic Lyme Disease	6	CPP, DEP, ANX, RA, NEURO, CVD	NEURO, MH, MSK, NEURO, CVD	0				
	Tuberculosis	10	DM, SUB, CLD, REN, VIS-I, HIV, CA-H, CKD, COG, MH	MET, MH, DIG, URO, VIS-I, INF, CA, URO	1				
