# Supplementary material for: Making clinical guidelines work for people with multiple long term conditions: analysis and recommendations from review of single condition guidelines
Source: BMJ Med. 2026 Feb 23;5(1):e001495. doi: 10.1136/bmjmed-2025-001495 (PMC12933760; doi:10.1136/bmjmed-2025-001495)
Supplement: online supplemental table 3 [file bmjmed-5-1-s004.pdf]

Supplementary Table 4

Example recommendations from NICE single-condition guidelines by recommendation type

Recommendation type	Guideline	Recommendation
Recommendations on treatment or management tailored to co-existing conditions/MLTC	Venous Thromboembolic disease	When offering anticoagulation treatment, take into account comorbidities
	Thyroid disorders	Consider starting levothyroxine at a dosage of 25 to 50 micrograms per day with titration for adults aged 65 and over and adults with a history of cardiovascular disease.
	Bipolar disorder	The committee recommend that bipolar disorder is managed solely in primary care, re-refer to secondary care if the following applies: suspected comorbid alcohol or drug misuse.
	Multiple sclerosis	Consider oral baclofen as a first-line drug treatment to treat spasticity in people with MS who have specific treatment goals such as improving mobility or easing pain and discomfort. Take into account any contraindications, comorbidities and the person's preferences
Recommendation on testing/diagnosing co-existing condition/MLTC in the presence of index condition	Abdominal Aortic Aneurism	Encourage men aged 66 or over to self-refer to the NHS AAA screening programme if they have not already been screened and they have any of the following risk factors: chronic obstructive pulmonary disease (COPD), coronary, cerebrovascular or peripheral arterial disease, family history of AAA, hyperlipidaemia, hypertension, they smoke or used to smoke.
	Epilepsy	Be alert to anxiety, other mental health difficulties and the risk of suicide in people diagnosed with epilepsy.
	Eating disorder	For people with an eating disorder and diabetes: use a low threshold for monitoring blood glucose and blood ketones
	Chronic kidney disease	When determining individual aspirational Hb ranges for people with anaemia of CKD, take into account: their symptoms and comorbidities.
Test for condition when specific co-existing condition is present	Peripheral arterial disease	Assess people for the presence of peripheral arterial disease if they have diabetes
	Diabetes	Offer intermittently scanned continuous glucose monitoring (is CGM, commonly referred to as 'flash') to adults with type 2 diabetes on multiple daily insulin injections if they have a condition or disability (including a learning disability or cognitive impairment) that means they cannot self-monitor their blood glucose by capillary blood glucose monitoring
	Cystic Fibrosis	Assess for cystic fibrosis and, when clinically appropriate, perform a sweat test (for children and young people) or a cystic fibrosis gene test (for adults) in people with any of the following: recurrent and chronic pulmonary disease, such as: recurrent lower respiratory tract infections, clinical or radiological evidence of lung disease (in particular bronchiectasis)
	Autism	Consider assessment for possible autism when a person has: a history of a neurodevelopmental condition (including learning disabilities and attention deficit hyperactivity disorder) or mental disorder.

Recognise how co-existing conditions/MLTC affect needs, symptoms, or medication for condition	COPD	Before starting LAMA+LABA+ICS, conduct a clinical review to ensure that: acute episodes of worsening symptoms are caused by COPD exacerbations and not by another physical or mental health condition
	Multiple sclerosis	Do not assume that the person's fatigue is always caused by MS. Assess for other causes and manage these or refer the person for management if indicated. Other causes of fatigue may include: illnesses, such as infections, anaemia and thyroid dysfunction, anxiety and depression
	Schizophrenia	If those whose illness has not responded adequately to treatment, consider other causes of non-response, such as comorbid substance misuse (including alcohol), the concurrent use of other prescribed medication or physical illness.
	Osteoporosis	Depression: Fracture risks appear to be increased in people taking antidepressant medications, particularly SSRIs. It is unknown whether these risks are due to changes in bone mass or due to an increased risk of falls.
Condition increases risk of other co-existing conditions or poorer outcomes	Chronic heart disease	Advise people without known diabetes that if they have had hyperglycaemia after an acute coronary syndrome, they are at increased risk of developing type 2 diabetes
	Obstructive sleep apnoea	COPD–OSAHS overlap syndrome occurs in people who have both chronic obstructive pulmonary disease (COPD) and obstructive sleep apnoea/hypopnoea syndrome (OSAHS). The combined effect of these conditions on ventilatory load, gas exchange, comorbidities and quality of life is greater than either condition alone.
	Parkinson's	Because people with Parkinson's disease may develop impaired cognitive ability, communication problems and/or depression, provide them with both oral and written communication throughout the course of the disease…
	Irritable bowel disease	IBD is a widely accepted risk factor for the development of colorectal cancer
Ensure equity in care and access for co-existing conditions	Chronic heart disease	Make cardiac rehabilitation equally accessible and relevant to all people after an MI, particularly people from groups that are less likely to access this service. These include people from black, Asian and minority ethnic groups, older people, people from lower socioeconomic groups, women, people from rural communities, people with a learning disability and people with mental and physical health conditions.
	Dementia	Ensure that people living with dementia have equivalent access to diagnosis, treatment and care services for comorbidities to people who do not have dementia.
	Eating disorder	Ensure that all people with an eating disorder and their parents or carers (as appropriate) have equal access to treatments (including through selfreferral) for eating disorders, regardless of: any physical or other mental health problems or disabilities.
	Autism	The autism strategy group should develop local care pathways that promote access to services for all autistic adults including people with coexisting physical and mental disorders (including substance misuse) and people with learning disability

Recommendation to provide information about, treat or refer for management of co-existing condition/MLTC	Peripheral artery disease	Offer all people with peripheral arterial disease oral and written information about their condition. Discuss it with them so they can share decision-making, and understand the course of the disease and what they can do to help prevent disease progression. Information should include: control of diabetes, hyperlipidaemia, diet, body weight and exercise, how they can access support for dealing with depression and anxiety.
	Hypertension	Discuss starting antihypertensive drug treatment, in addition to lifestyle advice, with adults aged under 80 with persistent stage 1 hypertension who have 1 or more of the following:<br>• target organ damage<br>• established cardiovascular disease<br>• renal disease • diabetes<br>• an estimated 10-year risk of cardiovascular disease of 10% or more.
	Peripheral neuropathy	When agreeing a treatment plan with the person, take into account their concerns and expectations, and discuss: the benefits and possible adverse effects of pharmacological treatments, taking into account any physical or psychological problems, and concurrent medications
	Prostate cancer	Help people decide whether to have an MRI or prostate biopsy by discussing: any comorbidities, together with their risk factors (including increasing age and black African–Caribbean family background).
Refer to relevant NICE guideline for managing co-existing conditions/MLTC	Heart valve disorders	If people have other indications for anticoagulation or antiplatelet therapy, follow the recommendations in the NICE guidelines on atrial fibrillation and acute coronary syndromes.
	Arrhythmia/Atrial fibrilation	Offer monitoring and support to modify risk factors for bleeding, including:<br>• uncontrolled hypertension (see NICE's guideline on hypertension in adults)<br>• harmful alcohol consumption (see NICE's guideline on alcohol-use disorders: diagnosis, assessment and management of harmful drinking and alcohol dependence)
	Chronic primary pain	Offer management options: in line with the NICE guideline for the underlying chronic pain condition if the underlying condition adequately accounts for the pain and its impact (see the NICE guidelines on headaches, low back pain and sciatica, rheumatoid arthritis, osteoarthritis, spondyloarthritis, endometriosis, neuropathic pain and irritable bowel syndrome).
	Depression	When the person has an anxiety disorder and comorbid depression or depressive symptoms, consult NICE guidance for the relevant anxiety disorder if available and consider treating the anxiety disorder first.

Use coordinated, multidisciplinary care for patients with MLTC	Cystic fibrosis	The specialist cystic fibrosis multidisciplinary team should either include or have access to specialist expertise relevant to cystic fibrosis in the following areas:<br>•microbiology<br>•pulmonary physiology<br>•diabetes<br>•gastroenterology<br>•hepatology<br>•rheumatology<br>•psychiatry<br>•interventional radiology<br>•surgery (gastrointestinal, thoracic, and ear, nose and throat)<br>•obstetrics<br>•palliative care.
	Lung cancer	Depression should be managed by multidisciplinary groups that include supportive and palliative care professionals
	Rheumatoid arthritis	Organise appropriate cross referral within the multidisciplinary team
	Tuberculosis	If the person has a comorbidity or coexisting condition such as: HIV or severe liver disease, for example, Child-Pugh level B or C or stage 4 or 5 chronic kidney disease (a glomerular filtration rate of <30 ml/minute/1.73m2) or diabetes or eye disease or impaired vision or pregnancy or breastfeeding or a history of alcohol or substance misuse, work with a specialist multidisciplinary team with experience of managing TB and the comorbidity or coexisting condition.
