# Supplementary material for: Making clinical guidelines work for people with multiple long term conditions: analysis and recommendations from review of single condition guidelines
Source: BMJ Med. 2026 Feb 23;5(1):e001495. doi: 10.1136/bmjmed-2025-001495 (PMC12933760; doi:10.1136/bmjmed-2025-001495)
Supplement: Supplementary file 5 [file bmjmed-2025-001495.reviewer_comments.pdf]

PEER REVIEW HISTORY

BMJ Medicine publishes all reviews undertaken for accepted manuscripts. Reviewers are asked to complete a checklist review form and are provided with free text boxes to elaborate on their assessment. These free text comments are reproduced below.

ARTICLE DETAILS

**TITLE (PROVISIONAL)**	Making clinical guidelines work for people living with multiple long-term conditions: Analysis and recommendations from a review of single-condition guidelines
**AUTHORS**	Pretorius, Sara; Bartle, Victoria; Bellass, Sue; Blackburn, Ella-Rose; Bold, Helen; Cooper, Rachel; Cordell, Heather; Evison, Felicity; Holding, Ray; Howe, Nicola; Lam, Emily; Marshall, Tom; Matthews, Fiona; Missier, Paolo; Pearson, Ewan; Plummer, Chris; Richards, Gillian; Robinson, Sian M; Sapey, Elizabeth; Scharf, Thomas; Singer, Mervyn; Suklan, Jana; Walker, Phakapan; Wason, James; Sayer, Avan; Witham, Miles

VERSION 1 - REVIEW

**REVIEWER NAME**	Chowdhury, Saifur R.
**REVIEWER AFFILIATION**	McMaster University, Department of Health Research Methods, Evidence, and Impact
**REVIEWER CONFLICT OF INTEREST**	None
**AI DISCLOSURE**	No
**DATE REVIEW RETURNED**	04-Apr-2025

**GENERAL COMMENTS**	Pretorius and colleagues conducted a review of existing guidelines on single conditions to examine how they addressed co-existing conditions and multiple long-term conditions or multimorbidity. This is an important study to reduce conflicting advice, increase the usefulness of guidelines for people living with multimorbidity, and reduce their treatment burden. Overall, the concept of the study is worthy and essential, given the novelty of its stand. The authors analyzed 68 guidelines covering 60 health conditions, with a focus on the UK healthcare context. The authors provided a descriptive summary of how single-condition guidelines considered co-existing and multiple long-term conditions in making their recommendations. 96% of guidelines provided a recommendation on diagnosis, treatment, or other management of the main medical condition in the presence of at least one other condition. 41% of guidelines provided clinical practice considerations for co-existing conditions. Only 22% of guidelines provided a specific reference to multiple long-term conditions and how patient care should be modified in the presence of specific multiple long-term conditions. Guidelines more frequently considered co-existing conditions within the same organ system than conditions in other organ systems when making recommendations. Guideline panels consist of larger members and more members with an interest in general internal medicine addressed co-existing conditions more extensively.<br><br>After carefully reading the whole manuscript, I feel that it would benefit if the authors addressed the following point in their revised version.

	1. The authors may revise the last sentence in the abstract conclusion. “there has been little progress toward a transformative approach…” the result of this study itself does not provide such a definitive conclusion, given that some of the guidelines reviewed could have been published before the prior recommendations were issued.<br>2. Please explain why the row total is not 68 in Table 1.<br>3. I think it’s appropriate to report the median instead of the mean throughout the manuscript, including tables since these are small numbers and not normally distributed.<br>4. Table 2, I did not understand how the authors calculated the proportion of condition combinations covered and how they got the numerator and denominator.<br>5. Similarly, “Of all the possible specific index condition / co-existing condition combinations, only a minority were addressed in surveyed guidelines (557/5236; 10.6%). There were significant differences between condition categories in the proportion of condition combinations covered, ranging from 21/539 for cancer to 101/770 for mental health (chi2 31.91, p <0.001). Of the 557 condition / co-existing condition combinations covered in guidelines, 132 (20%) were concordant combinations.” How was this combination generated?<br>6. How did the authors differentiate MLTC from comorbidities in supplementary file 3?<br>7. Supplementary table 3, a lot of information are presented, but these are difficult to follow. Without closely reading the methods section, the table alone does not clearly convey the meaning of the data presented.<br>8. Please provide a justification in the method section or in the supplementary file as to why the authors followed this 5-tier process to identify these guidelines and keep NICE and SIGN at the top tier and UK NHS trusts at the bottom tier.<br>9. Please include a description within Supplementary File 2. On its own, the table is difficult to interpret without referring to the explanation provided in the main text.<br>10. Define concordant and discordant conditions in the methods section.<br>11. Page 15, “Additionally, the NIHR HRC’s Insight Panel” elaborates on the term HRC.<br>12. In the result section - “Summary of inclusion of co-existing conditions and MLTC”, include percentages with the number to make it consistent with the abstract.<br>13. Supplementary file 3, at the top, is it necessary to use different colored tick marks to distinguish between guideline sources (e.g., NICE or SIGN)?

**REVIEWER NAME**	Sharifan, Amin
**REVIEWER AFFILIATION**	University for Continuing Education Krems, Department for Evidence-based Medicine and Evaluation
**REVIEWER CONFLICT OF INTEREST**	None
**AI DISCLOSURE**	No
**DATE REVIEW RETURNED**	12-Apr-2025

**GENERAL COMMENTS**	This manuscript presents a review examining the underrepresentation of multiple long-term conditions and co-existing conditions in UK-relevant clinical guidelines. While the

	recommendations provided are valuable, the manuscript has several issues that should be addressed. Overall, it lacks focus, alternating between multiple long-term conditions and co-existing conditions, with inconsistent narrative voice. Additionally, it falls short of meeting scoping review standards, as search methodology, databases used, and document retrieval processes are inadequately described. Below are detailed comments on each section.<br><br>a) Abstract:<br>1) The abstract should follow BMJ Medicine guidelines for original research, using structured headings: "Objective," "Methods and Analysis," "Results," and "Conclusion." The methods section is currently missing, and including "Participants" as a heading seems inappropriate for this type of study.<br>2) While the terminology aligns with the National Institute for Health Research framework, it may not be accessible to an international audience. Consider rephrasing the objective to improve clarity for global readers.<br>3) In the Results section, the focus on clinical guidelines for single conditions could imply applicability to only a niche population. Consequently, the meaning of "general medicine" in the final sentence requires clarification.<br>4) The first sentence overgeneralizes the findings; specify the UK setting (e.g., "Clinical guidelines within the UK..."). The abstract should conclude with forward-looking statements highlighting future research directions or practical implications.<br><br>b) Summary box:<br>1) The summary box contradicts the abstract regarding the investigation of co-existing conditions. The "What is already known" section should align with the abstract to avoid confusion.<br>2) This section overlaps substantially with the abstract. Consider expanding on unique aspects of the study (e.g., methodological nuances or broader implications) to provide additional value.<br><br>c) Introduction:<br>1) References 5 and 6 (global perspective) and 7 (older adults in England) create inconsistency in the epidemiological narrative. Ensure alignment between cited evidence and the scope of claims made.<br>2) The assertion supported by reference 24 does not clearly derive from the provided source. Please verify this citation or clarify the connection.<br>3) To better engage clinicians, include a concrete clinical example illustrating the challenges of managing multiple long-term conditions and co-existing conditions. The second paragraph lacks sufficient background on co-existing conditions—addressing this gap would strengthen the overall rationale.<br>4) Please clarify what distinguishes your study from previous work such as: 10.1513/pats.201208-063ST and https://www.bristol.ac.uk/primaryhealthcare/resources/pp4m/.<br>5) The text inconsistently switches between multiple long-term conditions and co-existing conditions. Streamline the narrative to maintain a consistent focus to ensure each concept is distinctly defined and contextualized.<br><br>d) Methods:<br>1) The setting mentioned (hospital) does not match the one described in the abstract (primary care, secondary care, tertiary care, community health settings, care homes, and remote settings).

	2) Based on my understanding, the study focused on guidelines relevant to sixty previously defined conditions applicable to UK practice. However, in the third paragraph of the methods section, you include five "descriptors," some of which (ophthalmic and congenital diseases) appear irrelevant to the diseases listed in supplementary table 1. Please elaborate on this inconsistency and the rationale behind it.<br>3) The data extraction section details how co-existing conditions were identified, while providing minimal information on multiple long-term conditions, which were extensively introduced in the previous section. It remains unclear whether the focus in data extraction was on co-existing conditions, multiple long-term conditions, or both.<br>4) Supplementary Tables 2 and 3 are not appropriately designed, making it difficult to evaluate. Consider registering your data in a public repository and sharing the link.<br>5) Regarding statistical analyses, the authors mention that continuous variables were analyzed with Student's t-test; however, no such data appears in the text or tables. If you refer to the means calculated based on categorical variables, please provide rationale for such an approach. Furthermore, the use of inferential statistics in this context is questionable. If the study aimed to capture all relevant guidelines for UK clinical practice, what is the purpose of making statistical inferences from a sample to a population? I would also strongly discourage using the term "trend" in significance testing.<br><br>e) Results:<br>1) When discussing an average number of conditions addressed by the guidelines (a dichotomous outcome), does presenting this as a decimal value make clinical sense?<br>2) The chi-squared test is typically limited to a two-by-two table. When discussing "differences between condition categories in the proportion of condition combinations covered," did you only compare cancer to mental health?<br><br>f) Reporting guideline:<br>1) If the study was meant to be a scoping review, why was the PRISMA 2020 checklist provided?

**REVIEWER NAME**	Laviolle, Bruno
**REVIEWER AFFILIATION**	Université de Rennes
**REVIEWER CONFLICT OF INTEREST**	None
**AI DISCLOSURE**	No
**DATE REVIEW RETURNED**	14-Apr-2025

**GENERAL COMMENTS**	Thank you for the opportunity to review this manuscript. In this paper, Pretorius et al. examine how clinical guidelines for specific single conditions address co-existing conditions.<br><br>While the topic is interesting and relevant, the manuscript requires substantial revisions before it can be considered for publication.<br><br>General Comments:<br>The Results section is difficult to follow due to the heavy reliance on supplementary tables. Supplementary Tables 2 and 3 are particularly problematic—they are unclear and appear to have missing elements.

	Major Comments:<br><br>Methods Section:<br><br>Page 14, line 17: Supplementary Table 2 is not displayed correctly and cannot be read properly.<br><br>Page 14, line 55: Please define the terms "concordant" and "discordant" conditions for clarity.<br><br>Page 15, line 8: Supplementary Table 3 also cannot be read properly.<br><br>Page 15, lines 8–30: This content may be better presented as a footnote to the table rather than in the main text.<br><br>Results Section:<br><br>The Results section is overly long and should be shortened to better align with the study objectives.<br><br>The subsection on concordant and discordant conditions includes statistical comparisons, but the text lacks a clear summary. A dedicated table showing these comparisons would improve clarity and allow readers to see exactly how many comparisons were conducted.<br><br>Page 17, line 50: It appears that the number of conditions is not normally distributed (mean = 3, SD = 2.5). In this case, reporting the minimum and maximum values may be more appropriate than using the standard deviation.

**REVIEWER NAME**	Raudasoja, Aleksi
**REVIEWER AFFILIATION**	Finnish Medical Society Duodecim
**REVIEWER CONFLICT OF INTEREST**	None
**AI DISCLOSURE**	No
**DATE REVIEW RETURNED**	17-Apr-2025

**GENERAL COMMENTS**	Thank you for the opportunity to review this manuscript.<br>Overall, it was a pleasure to read and show clear hard work.<br>The authors have taken a good approach to research the important issue of multimorbidity in guidelines. Especially, the recommendations show a clear way for improvement.<br>I hope my comments below help improve the manuscript.<br><br>Couple of overall comments:<br>1) I find it difficult to comprehend what the end goal is in this issue and how well the guidelines included reach those goals. It seems reasonable that guidelines should give consideration on multimorbidity or co-existing conditions when it is relevant for the index condition. Therefore, I would be more specific in what the end goal is, how many of the guidelines included in the end should be considering these issues. If authors think all the guidelines included should be considering these issues and mentioning them in the guideline, then provide more rationale for why this is the case.<br>2) Furthermore, it seems natural that concordant conditions are mentioned more often than discordant conditions as they are likely

	more important to the index condition. Therefore, it may be also good to find that guidelines are doing this, and it would be a bad sign if they mentioned these conditions as often (although they probably being not as relevant). Could authors elaborate on this more on this?<br><br>Below are my specific comments regarding the manuscript sections:<br><br>Abstract:<br>1) Abstract is difficult to follow as the headlines are not suitable for review methods. Abstract should follow the suitable sections from PRISMA guidelines for systematic review abstracts. For example, the following headlines may work better:<br>a. objectives<br>b. design – consider including statement on forming recommendations<br>c. methods - including eligibility criteria (for including guidelines), data extraction, synthesis, and forming of recommendations<br>d. results<br>e. conclusions<br>2) Your main outcome seems to be if and how guidelines provided considerations for concordant and discordant conditions – if so, this should be stated in the abstract. If it was something else, please clarify that. Currently it is hard to understand what specifically is evaluated in the review.<br>3) Since you aim to provide recommendations for improvement in the manuscript title, I would summarize the recommendations in the abstract. I also think the recommendations should be one of your key messages.<br><br>Introduction:<br>1) Please clearly state in the end that your aim was to provide recommendations for improvement as it appears to be.<br><br>Methods:<br>1) Consider providing separate paragraph for outcomes and explain “concordant conditions” and “discordant conditions” as well as other outcomes collected<br>a. it seems that all outcomes or variables collected are not yet reported (results section reports more outcomes/variables.<br>2) Provide the methods to form recommendations in the end of the methods section.<br><br>Results:<br>- row 39, page 15: Was guideline for spinal injury included or something related to that in which case please specify what was included.<br>Discussion<br>- Please specify what the good practices were at the end of first paragraph<br><br>Box 2:<br>- The recommendations feel relevant and actionable<br>- However, it is difficult to comprehend how the results of this review affected the results: were these methods identified from the previous guidelines, developed through discussion to address the current limitations or what? Please specify in the methods section. Please also clarify, where relevant, which specific result led to a specific recommendation.<br>- The last recommendation is difficult to understand.

	- Could you elaborate more in the discussion section on how these new methods (considering multimorbidity) could influence the work in the clinic. Also, how would they affect the workload. Recent research suggests guidelines are currently impossible to follow completely given the workload. Check e.g. Johansson M, Guyatt G, Montori V. Guidelines should consider clinicians' time needed to treat BMJ 2023; 380 :e072953 doi:10.1136/bmj-2022-072953

**REVIEWER NAME**	Schmidt-Mende, Katharina
**REVIEWER AFFILIATION**	Karolinska Institute, Neurobiologi, caring sciences and community health
**REVIEWER CONFLICT OF INTEREST**	None
**AI DISCLOSURE**	To make english more fluent
**DATE REVIEW RETURNED**	28-Apr-2025

**GENERAL COMMENTS**	Thank you for asking me to review this interesting manuscript aiming to understand more about the extent to which single-disease guidelines take into account MLTC. It is an interesting and clinically relevant research idea. The authors have reviewed 68 treatment guidelines on single diseases to assess to which degree they take into account the presence of comorbitities or MLTC. The authors conclude that guidelines still fail to take into account MTLC to a sufficient extent.<br>The paper is nicely written and easy to follow. I like especially box 2, as you summarize your findings and make concrete suggestions on how future guidelines could be improved, thus "bringing your findings a step further".<br>I have some suggestions for improvement.<br>Methods, table 2: I can´t see that the contents of table 2 are described in the methods section. I find it hard to understand the denominator in column "total"/concordant/disconcordant despite the description offered in the legend. Is there some way to describe this in a different and more comprehensive way? Likewise, I find it difficult to follow the results on page 17, lines 21.23. What do you mean by "of all the possible specific index conditions…..combinations". Please develop.<br>Table 2: explain "proportion of condition combinations covered" in methods, give an example, otherwise difficult to understand<br>Page 19, line 39: please explain why concordant conditions are the minority of co-existing conditions.<br>Page 20, lines 6-7: yes, I totally agree. As clinician, it is impossible to follow up on cross-references. Cross-referencing is not a sufficient way of "taking into account" MTLC.<br>Suppl table 3: is it possible to give more general information regarding the content of the table, as it is really difficult to get an overview. I would also recommend to highlight the MTLC section more (column I), as this is somewhat your main aim/finding. It is not easy to discover though as a reader.<br>Supplementary Table 4. Please rephrase the caption of the table, you describe more than "Numbers of guideline committee members by organ system"

**REVIEWER NAME**	Perera, Rafael
**REVIEWER AFFILIATION**	University of Oxford, Primary Care Health Sciences
**REVIEWER CONFLICT OF INTEREST**	I am an active researcher in the area of MLTCs and have received funding from the NIHR (UK) to study this area.

**AI DISCLOSURE**	I have used Grammarly to help with the spelling and grammar of the review. AI has not been used for anything else.
**DATE REVIEW RETURNED**	18-Jun-2025

**GENERAL COMMENTS**	bmjmed-2025-001495: Making clinical guidelines work for people living with multiple long-term conditions: analysis and recommendations from a review of single-condition guidelines<br><br>The current manuscript presents information from a review of clinical guidelines of single conditions (from a set of 60 long-term conditions) and explores how suitable these guidelines are for supporting the management of people living with multiple long-term conditions (MLTC). Their approach is UK-centric, and their way of identifying the guidelines to study showcases this, with most of the guidelines selected being developed by NICE. Their main findings are that most single-condition guidelines do not provide adequate guidance regarding the adjustments to management required in the presence of MLTC, but that some exemplary guidelines are better than the rest. These tend to have larger members in the writing group, with some evidence that generalists being present might improve their appropriateness for MLTCs.<br><br>Although the main message is likely useful, there are some aspects of the methods used and of the reporting that will require clarification and justification.<br><br>Theoretically, MLTC is different to co-morbidities. The latter term emphasises an index condition with others co-occurring. For MLTC, the focus is on a more holistic evaluation of the individual with no ‘index’ condition. Given that the review is based on guidelines for single conditions, it is understandable that the guidelines themselves will talk about co-morbidities, and this impacts the current article, where these two terms appear to be interchangeable. This will need some clarity in the Background and better specification of what the authors mean when they use these terms.<br><br>There are two large supplementary files that provide a wealth of data (Supplementary Tables 2 and 3). They were not adequately converted to the manuscript PDF, and it is easy to understand why this is when accessing this information. However, a reader will not be able to access these Excel files, and the amount of information (particularly for Supplementary Table 3) is so vast that it is difficult to digest. Further work is required to provide a table that could be converted to a PDF.<br><br>In the Data Analysis subsection (Methods Section), there is a section describing the use of t-tests and chi-squared for comparisons. Given that the sample is not ‘representative’ of anything (selection of the guidelines was determined and followed a precise algorithm), a formal comparison (e.g. hypothesis test) is meaningless. Descriptive statistics are sufficient in presenting the current state of the evidence in this area.<br><br>Regarding this last point, there is an overinterpretation of associations observed (e.g. more generalists in the panel, better representation of MLTC). It is unclear what is cause and what is effect so downplaying these associations would be appropriate.<br><br>Other general points:

	There are some numerical results that require explanation. For example, it is unclear how the different categories in Table 1 relate. If these are mutually exclusive or if there are just different ways of presenting data which are not related to each other. Similarly, the number of 'possible combinations = 5236' needs explaining.<br><br>The data extracted from each of the different guidelines (e.g. number of co-morbidities included) is unlikely to be normally distributed. Consider presenting these results using median and IQRs or justify your use of means and SDs.<br><br>The algorithm used for the identification of the different guidelines (five steps) will need some justification.

VERSION 1 – AUTHOR RESPONSE

	Reviewer	Section	Comment	Response
9	Reviewer 1		1. The authors may revise the last sentence in the abstract conclusion. "there has been little progress toward a transformative approach…" the result of this study itself does not provide such a definitive conclusion, given that some of the guidelines reviewed could have been published before the prior recommendations were issued.	Thank you for pointing this out. We agree that the original wording may have overstated the conclusions that can be drawn from our findings. On reflection, we feel that the final sentence did not accurately represent the conclusions of the study, and we have therefore revised it to more appropriately reflect our findings (p6).
10			2. Please explain why the row total is not 68 in Table 1.	The row total does not add up to the total number of guidelines because the categories are not mutually exclusive. A single guideline can provide recommendations that relate to a specific co-existing condition (e.g., stroke and cancer) and also include more general recommendations for treating, for example, stroke in the presence of various co-existing conditions. Therefore, a single guideline can be counted in both categories, meaning the sum of the rows will be higher than the total number of guidelines. We have narrowed our included guidelines to 56 NICE guidelines. We have therefore revised Table 1 to align with the new number of included guidelines and added a footnote to the table to explain the above.

11			3. I think it's appropriate to report the median instead of the mean throughout the manuscript, includining tables since these are small numbers and not normally distributed.	We have amended the manuscript to report medians (with interquartile range where appropriate) throughout
12			4. Table 2, I did not understand how the authors calculated the proportion of condition combinations covered and how they got the numerator and denominator.	We have now explained this in more detail in the Methods section (p 16 para 2) and in the Table legend
13			5. Similarly, "Of all the possible specific index condition / co-existing condition combinations, only a minority were addressed in surveyed guidelines (557/5236; 10.6%). There were significant differences between condition categories in the proportion of condition combinations covered, ranging from 21/539 for cancer to 101/770 for mental health (chi2 31.91, p <0.001). Of the 557 condition / co-existing condition combinations covered in guidelines, 132 (20%) were concordant combinations." How was this combination generated?	Again, we have now explained this calculation in more detail in the Methods section (p 16 para 2).
14			6. How did the authors differentiate MLTC from comorbidities in supplementary file 3?	Thank you for the comment. Recommendations were classified as relating to MLTC only when they explicitly referenced multimorbidity or synonymous terms such as "MLTC," "multimorbidity," or "multiple co-existing conditions." Recommendations that mentioned co-existing conditions more generally, without specific reference to MLTC, were not included in this category. This approach ensured that the MLTC-coded data reflected intentional guidance addressing multimorbidity rather than broader comorbidity contexts.
15			7. Supplementary table 3, a lot of information are presented, but these are difficult to follow. Without closely reading the	Supplementary Table 3 has been revised and streamlined to enhance clarity. The complete original matrix is available via the Newcastle

			methods section, the table alone does not clearly convey the meaning of the data presented.	University Figshare repository (DOI:10.25405/data.ncl.29911853).
16			8. Please provide a justification in the method section or in the supplementary file as to why the authors followed this 5-tier process to identify these guidelines and keep NICE and SIGN at the top tier and UK NHS trusts at the bottom tier.	We have reflected on this feedback (an issue which several reviewers and editors highlighted) and we have chosen now to limit inclusion of guidelines to NICE guidance to ensure that the selection process and inclusion of guidelines is robust, consistent and transparent. We have reworked all of the analyses to reflect this now.
17			9. Please include a description within Supplementary File 2. On its own, the table is difficult to interpret without referring to the explanation provided in the main text.	Supplementary File 2 has been updated to include illustrative examples for each recommendation type, and the title has been revised for greater clarity. The full original table, now more clearly titled, remains accessible via the Newcastle University Figshare repository (DOI:10.25405/data.ncl.29911853).
18			10. Define concordant and discordant conditions in the methods section.	Definitions of concordant and discordant conditions have been added to the abstract Results section (page 5, para 3) and to the methods section (p16 para 2).
19			11. Page 15, "Additionally, the NIHR HRC's Insight Panel" elaborates on the term HRC.	The full name of the NIHR HRC has been added on p17, para 3.
20			12. In the result section - "Summary of inclusion of co-existing conditions and MLTC", include percentages with the number to make it consistent with the abstract.	Percentages have been added to ensure consistency across sections.
21			13. Supplementary file 3, at the top, is it necessary to use different colored tick marks to distinguish between guideline sources (e.g., NICE or SIGN)?	Thank you for highlighting this. We have now confined our analysis to NICE guidelines only and therefore removed data relating to non-NICE guidelines from the matrix which is now accessible via the Newcastle University Figshare repository (DOI:10.25405/data.ncl.29911853).
22	Reviewer 2	Abstract	1) The abstract should follow BMJ Medicine guidelines for	Thank you for highlighting the structured headings for original research abstracts. The

			original research, using structured headings: "Objective," "Methods and Analysis," "Results," and "Conclusion." The methods section is currently missing, and including "Participants" as a heading seems inappropriate for this type of study.	headings have now been amended to align with these.
23		Abstract	2) While the terminology aligns with the National Institute for Health Research framework, it may not be accessible to an international audience. Consider rephrasing the objective to improve clarity for global readers.	The objective has been rephrased to make it clearer and more accessible for an international audience.
24		Abstract	3) In the Results section, the focus on clinical guidelines for single conditions could imply applicability to only a niche population. Consequently, the meaning of "general medicine" in the final sentence requires clarification.	We have revised this section, and the reference to “members with an interest in general medicine” now cites “generalist clinicians (e.g., general practitioners, general physicians, or others without a single-specialty focus).”
25		Abstract	4) The first sentence overgeneralizes the findings; specify the UK setting (e.g., "Clinical guidelines within the UK..."). The abstract should conclude with forward-looking statements highlighting future research directions or practical implications.	The wording of the objectives has been changed to reflect the aim of reviewing UK-based guidelines. Additionally, the concluding sentence has been amended to indicate how our study could inform future guideline development.
26		Summary box	1) The summary box contradicts the abstract regarding the investigation of co-existing conditions. The "What is already known" section should align with the abstract to avoid confusion.	We acknowledge the potential for confusion due to inconsistent messages and have made amendments in both the abstract and summary box to address this.
27		Summary box	2) This section overlaps substantially with the abstract. Consider expanding on unique aspects of the study (e.g., methodological nuances or	Thank you for this helpful suggestion. We have revised the section to reduce overlap with the abstract and have expanded the content to highlight unique aspects of the study, including

			broader implications) to provide additional value.	methodological nuances and broader implications, as recommended.
28		Introduction	1) References 5 and 6 (global perspective) and 7 (older adults in England) create inconsistency in the epidemiological narrative. Ensure alignment between cited evidence and the scope of claims made.	The text has been amended to provide clarity on which statistics are relevant for a global population and which relate to the UK.
29		Introduction	2) The assertion supported by reference 24 does not clearly derive from the provided source. Please verify this citation or clarify the connection.	Thank you for pointing out this referencing error. The source has been changed to one which more clearly supports the claim.
30		Introduction	3) To better engage clinicians, include a concrete clinical example illustrating the challenges of managing multiple long-term conditions and co-existing conditions.	As per this suggestion, we have included examples of how MLTC and co-existing conditions complicate clinical decision making and health care (p10, para 1).
			The second paragraph lacks sufficient background on co-existing conditions—addressing this gap would strengthen the overall rationale.	Additional information has been included to clarify the distinction between MLTC and co-existing conditions, and to explain how examining references to co-existing conditions in clinical guidelines helps assess their relevance to people with MLTC.
31		Introduction	4) Please clarify what distinguishes your study from previous work such as: 10.1513/pats.201208-063ST and https://www.bristol.ac.uk/primaryhealthcare/resources/pp4m/.	Thank you for highlighting these resources. Our work examines guidelines across many different conditions which distinguishes it from the highlighted work on COPD and also focuses specifically on the content of guidelines rather than attempting to be a guide in itself to clinical practice. As the reviewer correctly points out, there are ‘how-to’ resources that distil good clinical practice (e.g. in primary care illustrated by the weblink, but also from geriatric medicine practice); our aim was not to replicate such resources. We have clarified our aims under ‘what this study adds’ (p 7 para 2) and added some additional text to the Introduction to clarify this (p 11para 2).

32		Introduction	5) The text inconsistently switches between multiple long -term conditions and co-existing conditions. Streamline the narrative to maintain a consistent focus to ensure each concept is distinctly defined and contextualized.	We have reworked the Introduction to make the distinction between multiple long-term conditions and co-existing conditions clearer, and to show how comorbidity relates to the concept of MLTC for the purpose of this paper (p14 para 1).
33		Methods	1) The setting mentioned (hospital) does not match the one described in the abstract (primary care, secondary care, tertiary care, community health settings, care homes, and remote settings).	This list of settings has now been removed from the abstract which should address the inconsistency
34		Methods	2) Based on my understanding, the study focused on guidelines relevant to sixty previously defined conditions applicable to UK practice. However, in the third paragraph of the methods section, you include five "descriptors," some of which (ophthalmic and congenital diseases) appear irrelevant to the diseases listed in supplementary table 1. Please elaborate on this inconsistency and the rationale behind it.	Thank you for highlighting this inconsistency which occurred due to wording changes in supplementary table 1. The descriptors have now been given their original naming and the naming of one of the descriptors has been changed within the report to ensure alignment.
35		Methods	3) The data extraction section details how co-existing conditions were identified, while providing minimal information on multiple long-term conditions, which were extensively introduced in the previous section. It remains unclear whether the focus in data extraction was on co-existing conditions, multiple long-term conditions, or both.	This section has been amended to reflect that data extraction included references to MLTC (multimorbidity).
36		Methods	4) Supplementary Tables 2 and 3 are not appropriately designed, making it difficult to evaluate. Consider registering your data in a public repository and sharing the link.	We have reworked and condensed these supplementary tables to make them easier to understand. We have made the raw Excel data available via the Newcastle University data repository (DOI:10.25405/data.ncl.29911853).

37		Methods	5) Regarding statistical analyses, the authors mention that continuous variables were analyzed with Student's t-test; however, no such data appears in the text or tables. If you refer to the means calculated based on categorical variables, please provide rationale for such an approach. Furthermore, the use of inferential statistics in this context is questionable. If the study aimed to capture all relevant guidelines for UK clinical practice, what is the purpose of making statistical inferences from a sample to a population? I would also strongly discourage using the term "trend" in significance testing.	We have now removed formal statistical comparisons to address this point, which was also raised by another reviewer.
38		Results	1) When discussing an average number of conditions addressed by the guidelines (a dichotomous outcome), does presenting this as a decimal value make clinical sense?	We have reworked the figures throughout to present median values which should also address this point
39		Results	2) The chi-squared test is typically limited to a two-by-two table. When discussing "differences between condition categories in the proportion of condition combinations covered," did you only compare cancer to mental health?	We have now removed formal statistical comparisons to address this point, which was also raised by another reviewer.
40		Reporting guideline	1) If the study was meant to be a scoping review, why was the PRISMA 2020 checklist provided?	This is not a scoping review. We included the PRISMA checklist as the nearest available checklist for our study type but we agree it is not a perfect fit because our methods are not those of a systematic review or scoping review. The PRISMA checklist is no longer provided.

41	Reviewer 3	General comments	The Results section is difficult to follow due to the heavy reliance on supplementary tables. Supplementary Tables 2 and 3 are particularly problematic—they are unclear and appear to have missing elements.	We have reworked and simplified both of these tables so that they convert to PDFs and are more easily understandable. We have also simplified and reordered the Results section to make this easier to follow.
42		Methods	Page 14, line 17: Supplementary Table 2 is not displayed correctly and cannot be read properly.	We have simplified this table into one providing a small number of example recommendations for each recommendation type.
43		Methods	Page 14, line 55: Please define the terms "concordant" and "discordant" conditions for clarity.	We have now done this as suggested in the abstract (page 5, para 3) and to the methods section (p16 para 2).
44		Methods	Page 15, line 8: Supplementary Table 3 also cannot be read properly.	We have reworked and simplified this table so that it converts to a PDF and is more easily understandable.
45		Methods	Page 15, lines 8–30: This content may be better presented as a footnote to the table rather than in the main text.	Thank you for this comment. The page and line reference was not entirely clear to us and may reflect differences in formatting. If this was intended to refer to the detailed description of how we populated the matrix, this material has now been removed, as Supplementary File 3 has been simplified to provide a smaller and more accessible summary.
46		Results	The Results section is overly long and should be shortened to better align with the study objectives.	We have simplified and reordered the Results section to make this easier to follow
47		Results	The subsection on concordant and discordant conditions includes statistical comparisons, but the text lacks a clear summary. A dedicated table showing these comparisons would improve clarity and allow readers to see exactly how many comparisons were conducted.	We have now removed formal statistical comparisons as suggested by other reviewers.

48		Results	Page 17, line 50: It appears that the number of conditions is not normally distributed (mean = 3, SD = 2.5). In this case, reporting the minimum and maximum values may be more appropriate than using the standard deviation.	We have amended the manuscript to report medians (with interquartile range where appropriate) throughout
49	Reviewer 4	General comments	I find it difficult to comprehend what the end goal is in this issue and how well the guidelines included reach those goals. It seems reasonable that guidelines should give consideration on multimorbidity or co-existing conditions when it is relevant for the index condition. Therefore, I would be more specific in what the end goal is, how many of the guidelines included in the end should be considering these issues. If authors think all the guidelines included should be considering these issues and mentioning them in the guideline, then provide more rationale for why this is the case.	Thank you for this helpful comment. We agree that our original text did not sufficiently explain the end goal of the review. We have now clarified that our intention was to examine the extent to which guidelines considered co-existing conditions and MLTC, recognising that the degree of relevance may vary depending on the index condition. We have added text to explain why consideration of MLTC is important across all guidelines, even where co-existing conditions may appear less directly relevant, in order to support safe, coordinated, and patient-centred care. (p 11 para 2). We have also made clearer how well the guidelines we reviewed met this aim.
50			Furthermore, it seems natural that concordant conditions are mentioned more often than discordant conditions as they are likely more important to the index condition. Therefore, it may be also good to find that guidelines are doing this, and it would be a bad sign if they mentioned these conditions as often (although they probably being not as relevant). Could authors elaborate on this more on this?	Thank you for this insightful comment. We agree that concordant conditions are likely to be referenced more frequently than discordant conditions, as they are often more directly relevant to the index condition and its management. We have now clarified this point in the manuscript and highlighted that our findings do reflect this pattern. At the same time, we note that discordant conditions can also have important implications for care, particularly where treatment for one condition may exacerbate or interact with another, and therefore their relative neglect in guidelines represents a potential gap. (p 22 para 2). Furthermore, given that concordant conditions are those affecting the same organ or body system, and there are many organs, discordant conditions would be expected to form the majority of coexisting conditions in clinical practice.

51		Abstract	Abstract is difficult to follow as the headlines are not suitable for review methods. Abstract should follow the suitable sections from PRISMA guidelines for systematic review abstracts. For example, the following headlines may work better:<br>a. objectives<br>b. design – consider including statement on forming recommendations<br>c. methods - including eligibility criteria (for including guidelines), data extraction, synthesis, and forming of recommendations<br>d. results<br>e. conclusions	We have amended the headings to Objectives, Methods and Analysis, Results and Conclusion in line with this comment and another reviewer's recommendation
52		Abstract	Your main outcome seems to be if and how guidelines provided considerations for concordant and discordant conditions – if so, this should be stated in the abstract. If it was something else, please clarify that. Currently it is hard to understand what specifically is evaluated in the review.	The abstract objectives section has been reworded to better reflect the aims and focus of the review.
53		Abstract	Since you aim to provide recommendations for improvement in the manuscript title, I would summarize the recommendations in the abstract. I also think the recommendations should be one of your key messages.	We have now amended the Abstract and key messages to do this as suggested
54		Introduction	1) Please clearly state in the end that your aim was to provide recommendations for improvement as it appears to be.	We have now added this at the end of the Introduction as suggested (p12, para 1)
55		Methods	Consider providing separate paragraph for outcomes and explain "concordant conditions" and "discordant conditions" as well as other outcomes collected	Definitions of concordant and discordant conditions have been added to the abstract (p5, para 3) and in the method section (p 16, para 2)

			a. it seems that all outcomes or variables collected are not yet reported (results section reports more outcomes/variables.	To improve clarity, we have structured the key outcomes as a bullet-point list
56			Provide the methods to form recommendations in the end of the methods section.	We have now added this at the end of the Methods section (p 17 para 2 ‘Recommendations’)
57		Results	Was guideline for spinal injury included or something related to that in which case please specify what was included.	The spinal injury guideline was included, partially addressing the condition "Long-term musculoskeletal problems due to injury" from the 60-condition list that informed guideline selection.
58		Discussion	Please specify what the good practices were at the end of first paragraph	Thank you for this suggestion. The discussion has been slightly restructured. However, where we mention examples of good practice the section has been expanded to summarise what the good practices were (page 24, paragraph 2).
59		Box 2	The recommendations feel relevant and actionable	Thank you for this comment
60		Box 2	However, it is difficult to comprehend how the results of this review affected the results: were these methods identified from the previous guidelines, developed through discussion to address the current limitations or what? Please specify in the methods section. Please also clarify, where relevant, which specific result led to a specific recommendation.	We have described the methods for development of the recommendations in a new section of the methods (p17, para 2).<br>Within the discussion, where we summarise our recommendations, we have anchored each of the proposed improvements in the key findings from the results (p24 para 2)
61		Box 2	The last recommendation is difficult to understand.	We have simplified this text to make it easier to understand.
62		Box 2	Could you elaborate more in the discussion section on how these new methods (considering multimorbidity) could influence the work in the	Thank you for this comment and for the link to this relevant article. We have now included a paragraph in our discussion section reflecting on the implications of following guideline recommendations and guideline

			clinic. Also, how would they affect the workload. Recent research suggests guidelines are currently impossible to follow completely given the workload. Check e.g. Johansson M, Guyatt G, Montori V. Guidelines should consider clinicians' time needed to treat BMJ 2023; 380 :e072953 doi:10.1136/bmj-2022-072953	implementation for clinical practice and clinician time (p25 para 2)
63	Reviewer 5	Box 2	The paper is nicely written and easy to follow. I like especially box 2, as you summarize your findings and make concrete suggestions on how future guidelines could be improved, thus "bringing your findings a step further".	Thank you for this comment
64		Methods	Table 2: I can´t see that the contents of table 2 are described in the methods section. I find it hard to understand the denominator in column "total"/concordant/disconcordant despite the description offered in the legend. Is there some way to describe this in a different and more comprehensive way?	We have described the analysis for table 2 and amended the way we explain our calculations in the Methods section (p16 para 2) and in the Table legend
65		Results	Likewise, I find it difficult to follow the results on page 17, lines 21.23. What do you mean by "of all the possible specific index conditions…..combinations". Please develop.	We have amended the wording in our results section to clarify the meaning and provided a more comprehensive description of the calculation used to determine the number of possible index condition-co-existing condition combinations in the method section (p16 para 2).
66			Explain "proportion of condition combinations covered" in methods, give an example, otherwise difficult to understand	Hopefully the more comprehensive description of what this means and how we calculated it, in the method section (p16 para 2) has made this easier to understand.
67		Results	Page 19, line 39: Please explain why concordant	The wording of this part of the discussion has been changed to better reflect our findings and present clearer interpretations (page 22 para

			conditions are the minority of co-existing conditions.	2). Given that concordant conditions are those affecting the same organ or body system, and there are many organs, discordant conditions would be expected to form the majority of coexisting conditions in clinical practice.
68		Results	Page 20, lines 6-7: Yes, I totally agree. As clinician, it is impossible to follow up on cross-references. Cross-referencing is not a sufficient way of "taking into account" MTLC.	Thank you for this comment
69		Suppl table 3:	Is it possible to give more general information regarding the content of the table, as it is really difficult to get an overview. I would also recommend to highlight the MTLC section more (column I), as this is somewhat your main aim/finding. It is not easy to discover though as a reader.	Supplementary Table 3 has been revised and streamlined to enhance clarity. The complete original matrix is available via the Newcastle University Figshare repository (DOI:10.25405/data.ncl.29911853).
70		Supplementary Table 4.	Please rephrase the caption of the table, you describe more than "Numbers of guideline committee members by organ system"	Thank you for this helpful observation. In response, we have removed the table previously titled "Numbers of guideline committee members by organ system" from the supplementary materials. We now present general frequency data on the number of generalist committee members descriptively within the main text, without comparative analyses. This change reflects reviewer guidance to downplay this aspect of the findings and avoid overinterpretation
71	Reviewer 6	Background	Theoretically, MLTC is different to co-morbidities. The latter term emphasises an index condition with others co-occurring. For MLTC, the focus is on a more holistic evaluation of the individual with no 'index' condition. Given that the review is based on guidelines for single conditions, it is understandable that the guidelines themselves will talk about co-morbidities, and this impacts the current article,	We have reworked the Introduction to make the distinction between multiple long-term conditions and co-existing conditions clearer, and to show how comorbidity relates to the concept of MLTC for the purpose of this paper (p14 para 1).

			where these two terms appear to be interchangeable. This will need some clarity in the Background and better specification of what the authors mean when they use these terms.	
72		Supplementary files 2 and 3	There are two large supplementary files that provide a wealth of data (Supplementary Tables 2 and 3). They were not adequately converted to the manuscript PDF, and it is easy to understand why this is when accessing this information. However, a reader will not be able to access these Excel files, and the amount of information (particularly for Supplementary Table 3) is so vast that it is difficult to digest. Further work is required to provide a table that could be converted to a PDF.	Supplementary Tables 2 and 3 have been revised and streamlined to enhance clarity. The complete original matrices are available via the Newcastle University Figshare repository (DOI:10.25405/data.ncl.29911853).
73		Methods and results	In the Data Analysis subsection (Methods Section), there is a section describing the use of t-tests and chi-squared for comparisons. Given that the sample is not 'representative' of anything (selection of the guidelines was determined and followed a precise algorithm), a formal comparison (e.g. hypothesis test) is meaningless. Descriptive statistics are sufficient in presenting the current state of the evidence in this area.	We have removed formal statistical comparisons as suggested
74		Results/discussion	Regarding this last point, there is an overinterpretation of associations observed (e.g. more generalists in the panel, better representation of MLTC). It is unclear what is cause and what is effect so downplaying these associations would be appropriate.	In response to reviewer feedback, we have removed all statistical tests, including correlational analyses. While we continue to present frequency data on the number of 'generalist' committee members, this information is discussed in a more descriptive and cautious manner within the discussion section. Furthermore, we explicitly acknowledge in the limitations that any

				inferences drawn from these data should be interpreted with caution (p26 para 1)
75		Results	There are some numerical results that require explanation. For example, it is unclear how the different categories in Table 1 relate. If these are mutually exclusive or if there are just different ways of presenting data which are not related to each other.<br>Similarly, the number of 'possible combinations = 5236' needs explaining.	We have added a footnote to the table to explain that categories are not mutually exclusive. A single guideline can provide recommendations that relate to a specific co-existing condition (e.g., stroke and cancer) and also include more general recommendations for treating, for example, stroke in the presence of various co-existing conditions. Therefore, a single guideline can be counted in both categories, meaning the sum of the rows will be higher than the total number of guidelines.<br><br>We have also provided a more comprehensive description of how the number of possible combinations was calculated (p16, para 1 and 2)
76		Methods/results	The data extracted from each of the different guidelines (e.g. number of co-morbidities included) is unlikely to be normally distributed. Consider presenting these results using median and IQRs or justify your use of means and SDs.	We have amended the manuscript to report medians (with interquartile range where appropriate) throughout
77		Methods	The algorithm used for the identification of the different guidelines (five steps) will need some justification.	We have reflected on this feedback and we have chosen now to limit inclusion of guidelines to NICE guidance to ensure that the selection process and inclusion of guidelines is robust, consistent and transparent. We have reworked all of the analyses to reflect this.
				Thank you for this helpful point. We agree that improving care for people with MLTC requires more than adapting guidelines, and also depends on factors including medical training, research, and health service structures. We have added text to the discussion to acknowledge this broader context, while noting that guidelines remain an important lever given their influence on clinical standards, service design, and professional education (p 26, para 2).

				We have also expanded the detail in Box 1 to give more specific examples from selected guidelines to illustrate good practice.
				Thank you for this comment. We agree that cross-referencing between guidelines during a consultation is unlikely to be feasible in many clinical settings. That said, your comment highlights an important point about usability and workload, which we have now addressed in our discussion (p25 para 2). Specifically, we note that guidelines may be difficult to implement in practice if they become overly complex, and we suggest that multimorbidity-related content should be integrated in ways that promote clarity, prioritisation, and practical application (e.g., through structured summaries and decision aids). We have also framed our recommendations in terms of guideline development processes -committee composition, evidence review, structure, and scope, rather than simply expanding content, to avoid adding to cognitive and administrative burden.
				We have simplified and reordered the Results section to address this point
				We have carefully considered this feedback and the feedback from the reviewers, and in light of this, we have reworked all of the analysis focussing solely on NICE guidelines to ensure that our approach is consistent

VERSION 2 – REVIEW

**REVIEWER NAME**	Laviolle, Bruno
**REVIEWER AFFILIATION**	Université de Rennes
**REVIEWER CONFLICT OF INTEREST**	None
**AI DISCLOSURE**	No
**DATE REVIEW RETURNED**	09-Oct-2025

**GENERAL COMMENTS**	Authors have adequately addressed my comments in the revised version of the manuscript. Therefore, I have no further comments.

**REVIEWER NAME**	Raudasoja, Aleksi
**REVIEWER AFFILIATION**	Finnish Medical Society Duodecim
**REVIEWER CONFLICT OF INTEREST**	None
**AI DISCLOSURE**	No
**DATE REVIEW RETURNED**	08-Oct-2025

**GENERAL COMMENTS**	Thank you for the another opportunity to review this article.<br><br>Authors have well addressed the comments that I had on the previous round.<br><br>I have a couple of remaining comments:<br>1. Discordant and concordant conditions should be defined in the same section than other outcomes.<br>2. Consider adding the proportion of guidelines considering discordant and concordant conditions in the abstract

**REVIEWER NAME**	Schmidt-Mende, Katharina
**REVIEWER AFFILIATION**	Karolinska Institute, Neurobiologi, caring sciences and community health
**REVIEWER CONFLICT OF INTEREST**	None
**AI DISCLOSURE**	No
**DATE REVIEW RETURNED**	12-Oct-2025

**GENERAL COMMENTS**	I am satisfied with the changes the authors have made and have no further suggestions for changes.

**REVIEWER NAME**	Perera, Rafael
**REVIEWER AFFILIATION**	University of Oxford, Primary Care Health Sciences
**REVIEWER CONFLICT OF INTEREST**	My research partially focuses on MLTCs and I have received funding from the UK NIHR to support my time and that of my team to research this area.
**AI DISCLOSURE**	No
**DATE REVIEW RETURNED**	23-Oct-2025

**GENERAL COMMENTS**	The authors have replied adequately to the comments and queries made by the editors and the reviewers. I believe the current version of the manuscript is suitable for publication in BMJ Medicine.

VERSION 2 – AUTHOR RESPONSE

	Reviewer	Section	Comment	Response

10	Reviewer 4	Methods	Discordant and concordant conditions should be defined in the same section than other outcomes.	Thank you for your comment regarding the placement of definitions for concordant and discordant conditions. We weren't entirely sure which section you were referring to, but we assume you meant the abstract, where these terms were previously defined in the results section rather than in the methods. To improve clarity and consistency, we've now moved the definitions to the methods section alongside other key outcome definitions
11		Abstract	Consider adding the proportion of guidelines considering discordant and concordant conditions in the abstract	Thank you for this suggestion. We have added percentages for the possible concordant and discordant condition combinations covered by the guidelines, enabling readers to better understand the proportion of each type represented in the analysis.
